# Chemoresistance and targeted therapies in ovarian and endometrial cancers

**DOI:** 10.18632/oncotarget.14021

**Published:** 2016-12-19

**Authors:** Kevin Brasseur, Nicolas Gévry, Eric Asselin

**Affiliations:** ^1^ Research Group in Cellular Signaling, Department of Medical Biology, Canada Research Chair in Molecular Gyneco-Oncology, Université du Québec à Trois-Rivières, Trois-Rivières, Québec, Canada; ^2^ Département de Biologie, Faculté des Sciences, Université de Sherbrooke, Boulevard de l’Université, Sherbrooke, QC, Canada

**Keywords:** gynecological cancers, chemoresistance, targeted therapies, PI3K, estrogen

## Abstract

Gynecological cancers are known for being very aggressive at their advanced stages. Indeed, the survival rate of both ovarian and endometrial cancers is very low when diagnosed lately and the success rate of current chemotherapy regimens is not very efficient. One of the main reasons for this low success rate is the acquired chemoresistance of these cancers during their progression. The mechanisms responsible for this acquired chemoresistance are numerous, including efflux pumps, repair mechanisms, survival pathways (PI3K/AKT, MAPK, EGFR, mTOR, estrogen signaling) and tumor suppressors (P53 and Par-4). To overcome these resistances, a new type of therapy has emerged named targeted therapy. The principle of targeted therapy is simple, taking advantage of changes acquired in malignant cancer cells (receptors, proteins, mechanisms) by using compounds specifically targeting these, thus limiting their action on healthy cells. Targeted therapies are emerging and many clinical trials targeting these pathways, frequently involved in chemoresistance, have been tested on gynecological cancers. Despite some targets being less efficient than expected as mono-therapies, the combination of compounds seems to be the promising avenue. For instance, we demonstrate using ChIP-seq analysis that estrogen downregulate tumor suppressor Par-4 in hormone-dependent cells by directly binding to its DNA regulatory elements and inhibiting estrogen signaling could reinstate Par-4 apoptosis-inducing abilities. This review will focus on the chemoresistance mechanisms and the clinical trials of targeted therapies associated with these, specifically for endometrial and ovarian cancers.

## INTRODUCTION

Gynecological cancers are pathologies developing in the women’s reproductive organs, mainly located in the uterus and ovaries. Overall, these cancers accounts for more than 10% of cancer deaths and new cases among women, each year in North America and Europe [1–6].

**Figure 1:**
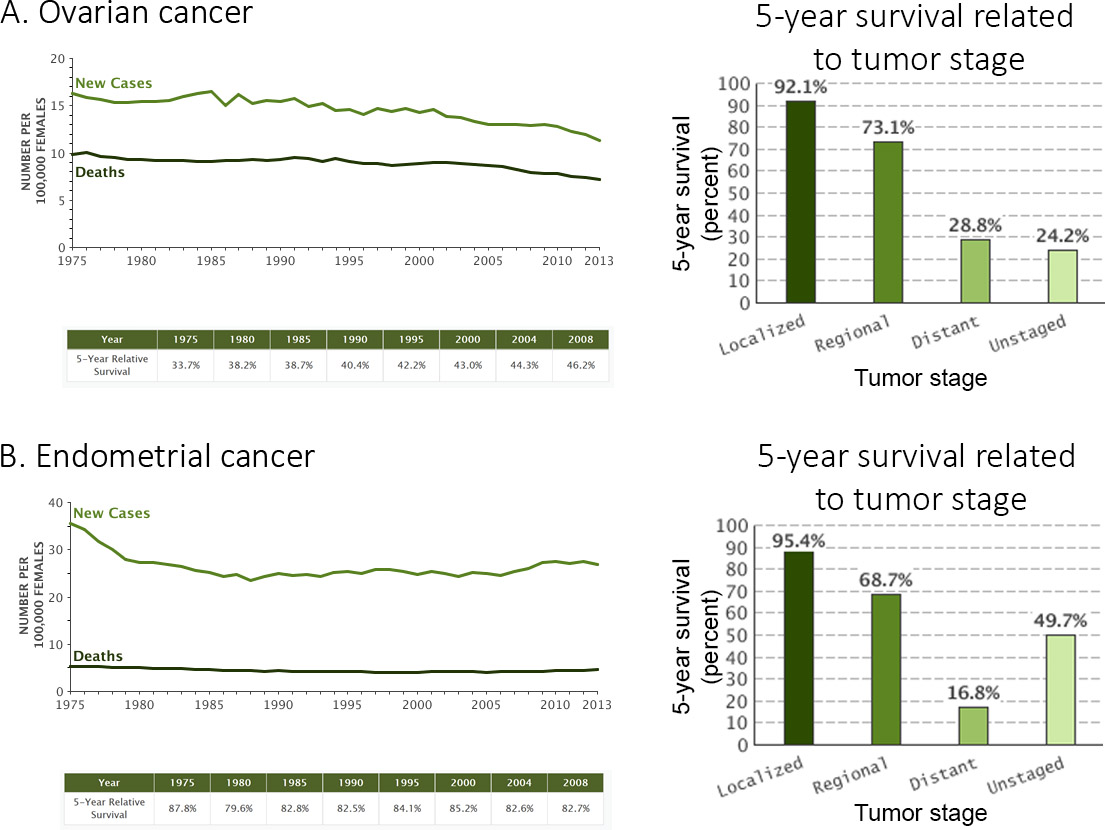
Ovarian and endometrial cancers statistics **A**. Ovarian and **B**. endometrial cancers statistics for new cases, deaths and 5-year survival). Data for new cases and deaths are represented by the number per 100 000 females (1975 to 2013), the 5-year survival rate (%) is available for all diagnosed patients (1975-2008) in a table or specifically sorted by tumor stage (2006-2012) in a histogram. The tumor stage is a factor related the chemoresistance. Data were obtained from seer.cancer.gov.

Ovarian cancer is hard to diagnose because of the almost total lack of symptoms during the early development stages of the tumor. Considering that more than 75% of the cases are detected at an advanced stage, ovarian cancer has a high mortality rate being the gynecological cancer with the lowest average 5-year survival rate (46%) (Figure 1A) [1–7]. An important fact to consider about ovarian cancer, as well as its low survival rate, is the current treatments low efficiency; current treatments become ineffective after a few cycles of administration, with a risk of recurrence estimated at 80-85% [4, 8].

Uterine cancer is the most frequent gynecological cancer and is frequently diagnosed early leading to a better outcome for the patient [1–5, 9]. Most of the cancers occurring in the uterus begin in the endometrium ( > 95%) and this subtype is called endometrial cancer [5]. Considering this fact, endometrial cancer will be mainly discussed here. Although the prognosis of endometrial cancer is good, more than 25% of patients are diagnosed at an advanced stage (Stage > 1) with an invasive primary tumor and subsequently accompanied by metastases [10]. One considerable hurdle for these patients diagnosed with an advanced/recurrent cancer, even though they are treated with aggressive therapies, is that the survival rate is very low ( < 20%) (Figure 1B) [5].

In the last decades, treatments for gynecological cancers have not much advanced beyond the platinum-based chemotherapy when compared with many other types of cancer; nor has the patient survival and cure rates increased much (Figure 1) [5, 6, 11, 12]. Reasons for the low survival outcome of gynecological cancers diagnosed lately/recurrent are the resistance to chemotherapy acquired by cancer cells and the non-selectivity of current treatments. This problematic lead to highly damageable side effects for the patients, thus limiting the use of drugs and how they are administered. Because of the inefficacy of the current chemotherapeutic regimens, more research and improvement of the current treatments are required to overcome this challenge.

In this manuscript, we will review the current treatments, their limitations against gynecological cancers and the molecular pathways responsible for the acquired chemoresistance thus leading to the current use of targeted therapies in clinical trials to increase the efficiency of treatments, which prevents recurrent cancers and increases survival of women suffering from these types of cancers.

## CURRENT TREATMENTS

Current treatments for both ovarian and endometrial cancers are known for being very similar. Initially, surgery is conducted in order to remove the vast majority of the tumor localized in its corresponding organ. Concerning advanced cancers, the remaining mass following the initial surgery is a good prognostic for survival. If needed, further treatments need to be administered to completely eliminate the tumor left and its distant metastases depending on the stage of the cancer [5, 13–15].

Radiation and hormone therapies are two potential methods of elimination of the remaining cancer cells, which can be used in both types of gynecological cancers. These two types of treatment are rarely used for ovarian and endometrial cancers when compared with breast tumors.

Concerning radiation, this method is rarely used as a treatment for ovarian cancer, considering their frequently late diagnosis, but is instead used as an option for recurrent cases, patients with high-risk of surgical mortality or those who cannot tolerate chemotherapeutic compounds. In the case of endometrial cancer, radiation is more frequently used considering the early diagnosis of the tumor; however, it is not as much used for advanced stages cancers [5, 14, 15].

Concerning hormonal therapies, a majority still expresses the estrogen receptors (mainly ERα) and progesterone receptor and those requiring hormones for growth can be classified as hormone dependent. Interestingly, hormone therapy has been used mainly to treat breast cancers and their effects are well known, but are sometimes prescribed in gynecological cancers despite the variability of their response rates [16, 17]. Not unlike breast cancer, mostly in the late stages, gynecological cancers can have mutations/inactivation leading to a loss of the expression of these hormone receptors or not responding to hormonal signals, becoming hormone-independent, thus making hormonal therapies ineffective [16, 18–21]. The different treatment administered for hormone therapy in gynecological cancers still expressing the ER consists of progestin, Luteinizing hormone-releasing hormone (LHRH) agonists and aromatase inhibitors [5, 14–17, 22–24]. The response rate of these types of treatment is moderate and they are mainly used for endometrial cancers expressing the ER. Notably, the success rate of hormonal therapy in gynecological cancers needs to be further investigated considering that many factors (receptor status, cancer stage, chemoresistance status, heterogeneity of the patients and drugs combination) can influence the efficiency of these treatments and were not always considered when used as a treatment in past studies [16, 17].

Lastly, the most frequently used method to eliminate the remaining gynecological cancer cells, widespread in the patient, is the chemotherapy approach. The principle of this method is to use anti-cancer drugs which generally target cells undergoing rapid division, a characteristic of cancer cells. The different chemotherapeutic drugs used for gynecological cancer consist mainly of platinum compounds (cisplatin or carboplatin), taxanes (paclitaxel or docetaxel) and doxorubicin [5, 10, 25]. Platinum compound mechanism consists of damaging DNA by forming platinum-DNA adducts leading to the inhibition of DNA replication and leading cells to apoptosis [26]. Taxanes mechanism is different and instead target microtubule polymerization, inhibiting mitosis and thus inducing apoptosis [27, 28]. Doxorubicin is an anthracycline compound which intercalate DNA, inhibits topoisomerase-II by stabilizing its complex and generate free radicals leading to cell death [29]. Some other chemotherapeutics drugs can also be helpful and used in gynecological cancers including cyclophosphamide (an alkylating agent), gemcitabine (a nucleoside analog), topotecan (a topoisomerase-I inhibitor) or vinorelbine (an inhibitor of mitosis through interaction with tubulin). These agents are mostly used in combination and the platinum-paclitaxel and platinum-doxorubicin combos have been designated as first-line treatment for gynecological cancers [5, 10, 30]. The response rate of these combinations is very good, being around 70% for ovarian cancer and 45% for endometrial cancer [5, 30]. However, a very frequent occurrence in gynecological cancers is that most of the patients relapse and the arising tumor becomes resistant to chemotherapeutic compounds, leading to a low survival rate [31, 32]. Overall, chemotherapy is a very efficient initial treatment but the recurrence of gynecological cancers and their acquisition of chemoresistance is a huge hurdle to overcome.

## CHEMORESISTANCE IN GYNECOLOGICAL CANCERS

Chemoresistance is presumably responsible for causing treatment failure and mortality for more than 90% of patients with cancer of advanced stage [13, 33]. This major hurdle, having an impact on patient survival, can be acquired via diverse modifications including the increase of efflux pumps to reject drugs and a decrease in cell division limiting the effect of chemotherapeutic compounds targeting mitosis arrest. At the molecular level, genes can be modified to influence the efficiency of repair proteins and diverse survival pathways while decreasing the level of different tumor suppressor. The following will discuss about the literature of known mechanisms of chemoresistance exclusively in gynecological cancers. A better understanding of these mechanisms will allow more efficient therapies to be administered to patients in the clinic.

### Efflux pumps

Overexpression of the multidrug-resistance gene MDR1 is associated with acquisition of chemoresistance, particularly against paclitaxel. This acquisition is explained by the increased level of the efflux pump P-glycoprotein (Pgp), thus eliminating more efficiently the presence of chemotherapeutic drugs in both endometrial and ovarian cancers [34–37]. Resistance to platinum compounds has also been related to increased levels of copper pumps [38–42]; indeed, it has been demonstrated that cisplatin-resistant ovarian cancer cell lines have acquired, in part, their resistance *via* an increased protein level of copper-transporting ATPases (ATP7A and ATP7B) [38, 42, 43]. In a patient-derived gene expression profile, ATP7B has also been associated as a chemoresistance marker in ovarian carcinomas treated with cisplatin [39]. Concerning endometrial cancer, copper-transporter ATP7B overexpression in endometrial carcinoma is also related to cisplatin resistance and indicate an unfavorable outcome for patients [40].

### DNA repair mechanisms

For a long time, mechanisms of DNA repair have been associated with chemoresistance in ovarian cancers [44–47].

#### Nucleotide excision repair process (NER)

One known mechanism responsible for the repair of platinum DNA adducts in ovarian cancer is the nucleotide excision repair process (NER) [48–51]. NER is a multi-step process implicating various proteins to remove and replace a sequence of nucleotides on a DNA strand. Enhanced NER is associated with increased resistance in ovarian cancer. The protein ERCC1, forming an endonuclease complex with XPF and involved in the 5′ incision of DNA adducts, has been reported to be correlated in the degree of sensitivity to platinum compounds in ovarian cancers [48–52]. XPF and XPG proteins, involved in NER process, are also reported to have an impact on platinum sensitivity of ovarian cancers [53]. On the contrary, very little association have been drawn between endometrial cancer and NER.

#### Mismatch repair (MMR)

Another repair mechanism, mismatch repair (MMR), is also known to be associated with chemoresistance mechanisms of ovarian cancers. The principle of MMR is to recognize a mismatched or unmatched DNA base, repair and reassemble DNA correctly [54]. When platinum compounds are administered, the MMR process is unable to complete repairs of mismatched DNA, thus leading to apoptosis [55]. It is suggested that a MMR deficiency in ovarian cancers, mainly due to the loss of the MLH1 gene, allows the cells to continue proliferating, even in presence of cisplatin or carboplatin, thus enabling chemoresistance through the failure to enter apoptosis following exposure to chemotherapy [56–61]. Conversely, other studies seems to report that there is no significant association between MMR deficiency and resistance to platinum compounds [62, 63]. They suggest that the limited quantity of samples studied and the presence of other potential resistance mechanisms could explain the absence of a significant association with MMR and platinum resistance. Very little has been studied concerning chemoresistance and MMR deficiency in endometrial cancers. Few studies report the acquisition of chemoresistance associated with MMR *via* the use of HEC59 endometrial cancer cell line [60, 64, 65]. Interestingly, endometrial cancer frequently has MMR deficiency associated with microsatellite instability which could have an impact on the efficiency of platinum compounds [66–69].

#### Homologous recombination (BRCA1/2 genes)

BRCA1 and BRCA2 are a known genes involved in an error-free repair mechanism *via* homologous recombination for double strand DNA breaks [70]. These genes are well known for increasing risks of breast as well as ovarian cancers when mutated and transmitted through by heredity [71–75]. Interestingly, mutations on BRCA1 and BRCA2 genes have also been associated with an increased risk of endometrial cancer, but this relation was observed more frequently in association with tamoxifen-treated women’s [76–78]. Downregulation of BRCA1 is frequent ( > 72%) in high-grade ovarian cancers [79, 80]. It was also observed with BRCA genes that they are involved in response to various chemotherapeutic drugs and consequently associated to chemoresistance [80]. Downregulation of BRCA1 in ovarian cancer provides sensitivity to platinum compounds while providing resistance to taxane drugs [80–85]. BRCA2 has also been associated with sensitivity to platinum compounds when mutated/downregulated in ovarian cancer [85, 86].

### Survival pathways

Survival pathways play a major role in mechanisms of chemoresistance of gynecological cancers.

#### PI3K/AKT pathway

**Figure 2:**
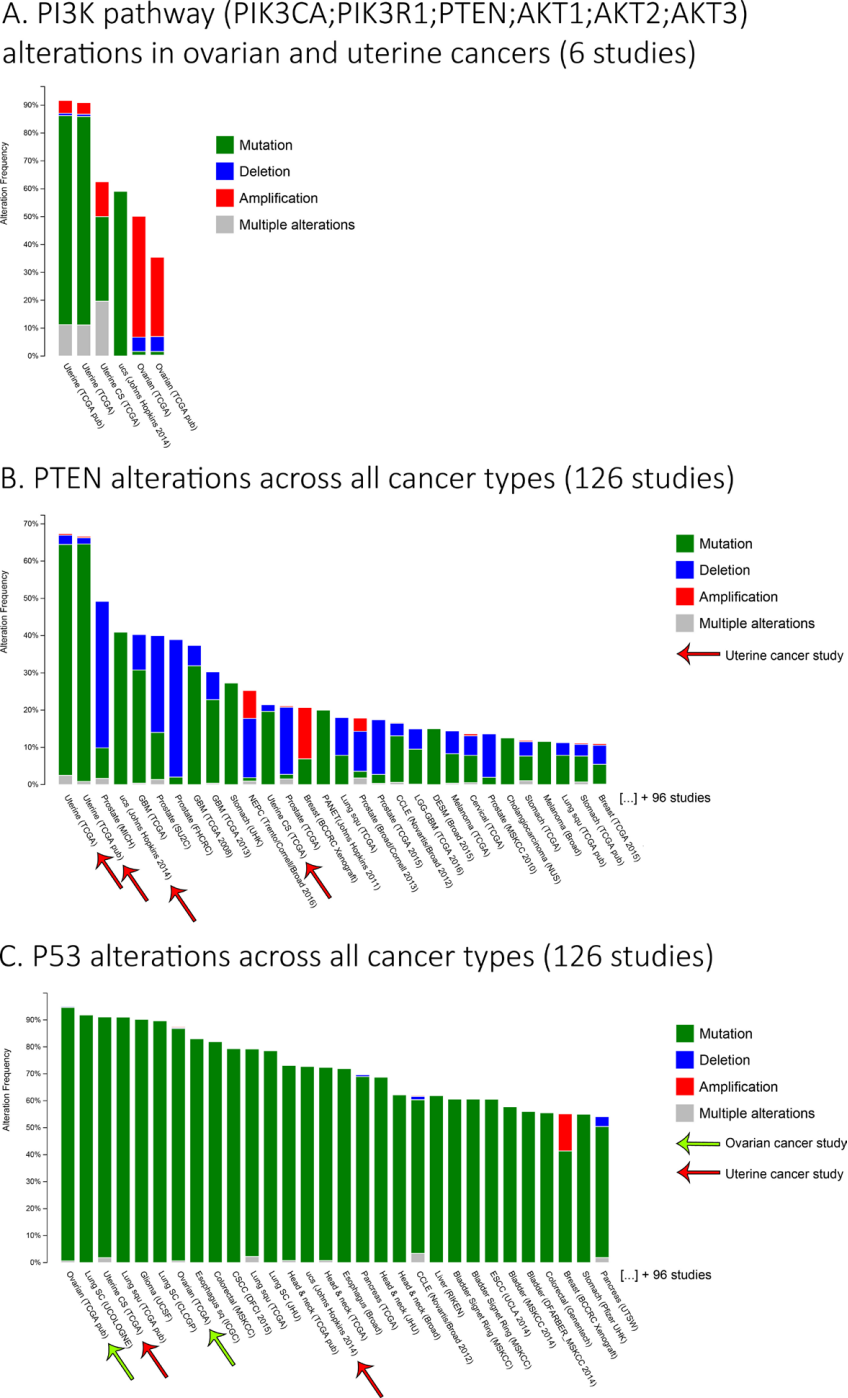
Ovarian and uterine cancers major alterations **A**. A histogram representing the frequency of alterations for genes from the PI3K pathway (PIK3CA, PIK3R1, PTEN, AKT1-2-3), specifically in ovarian and uterine cancers from 6 studies. **B**.-**C**. The histograms representing the frequency of alterations for **B**. PTEN or **C**. P53 in various cancer types from 126 studies. Only the first 30 studies are shown to first simplify the figure, but also to indicate the importance of these alterations in ovarian and uterine cancers. The studies shown in the histograms were sorted from those with the highest to the lowest frequency of alterations for the associated genes. Data were obtained using www.cbioportal.org database.

The PI3K/AKT survival pathway is one major signaling cascade, which is frequently mutated/hyperactivated at different levels in both ovarian and endometrial cancers [12, 87–91]. Using TCGA datasets, it is possible to observe that major components of the PI3K/AKT pathway present a high frequency of alteration in gynecological cancers ( > 40% in ovaries; > 90% in the uterus) (Figure 2A) [92, 93]. These alterations of the PI3K pathway are involved in the tumorigenesis of gynecological tumors but also their chemoresistance profile. PI3K is a kinase located on the cellular membrane, stimulated by growth hormones and responsible for phosphorylating PIP2 to PIP3. Once phosphorylated, PIP3 can activate downstream targets of the PI3K pathway such as AKT and PDK1 kinases, thus activating various downstream targets involved in protein synthesis and cell growth. PI3K and its subunits (mainly PIK3CA) are known for being highly mutated and responsible for increasing chemoresistance in ovarian cancer [94, 95]. Downstream of PI3K, AKT isoforms (AKT1-2-3) have been reported to also increase the chemoresistance against platinum drugs, taxane and doxorubicin, in both ovarian and endometrial cancers [95–104]. It has been demonstrated that only AKT1 and AKT2 isoforms are responsible for the acquisition of resistance against cisplatin and paclitaxel while all three isoforms of AKT increase doxorubicin resistance in endometrial cancer cells [98]. Concerning ovarian cancer, it has been demonstrated that AKT2 expression increase resistance to cisplatin [105]. PTEN is a tumor suppressor lipid phosphatase acting negatively on the PI3K pathway *via* its ability to dephosphorylate PIP3 to PIP2, thus controlling the activity of PI3K downstream targets. A very interesting fact concerning PTEN is the high percentage of alterations observed in endometrial cancers ( > 65%), which is astonishing when compared to other cancers types (Figure 2B) [92]. Observations have been made concerning this protein, PTEN, and chemoresistance status of gynecological cancers. Indeed, downregulation/inactivity of PTEN (frequently mutated in the endometrium) leads to an increase of resistance against platinum compounds [91, 94, 106–110]. XIAP, an inhibitor of apoptosis, is involved in PI3K/AKT pathway to protect cells by acting as a promoter of AKT activity *via* its interaction with PTEN as an E3 ubiquitin ligase, thus regulating negatively PTEN protein level and its cytosolic/nuclear localization [100, 111, 112]. XIAP is also involved in the chemoresistance against cisplatin (endometrial and ovarian), taxane (ovarian) and doxorubicin (endometrial) for both cancers [97, 100, 113–118]. P53 is another well-known tumor suppressor involved in the development of resistance observed in relation to the PI3K/AKT pathway in gynecological cancers. Indeed, it has been demonstrated that P53 inhibits PI3K activity, and consequently AKT, by binding on one of PIK3CA gene promoters thus inhibiting its transcription in ovarian cancer [119]. AKT can also, inversely, inhibit P53 activation through MDM2 and thus inhibit mitochondrial P53-dependent apoptosis [101, 102, 120]. Wild-type P53 is involved in the chemoresistance attributed to PI3K/AKT and XIAP in ovarian cancer. To overcome this resistance *via* the inhibition of PI3K pathway components, the presence of wild-type P53 is required for an optimal sensitization of the cancer cells [99, 101, 102, 118, 120].

#### MAPK pathway

Another survival pathway to consider in gynecological cancers is the MAPK pathway. MAPK pathway consists of cascades of protein kinases, which can be activated by various stimuli including growth factors or genotoxic stress. Following stimulation, MAPK play a major role for cell growth, survival and/or apoptosis. An important aspect of the MAPK pathway is the fact that upstream of one cascade lies the RAS oncogene, frequently deregulated in various cancers including those affecting gynecological tissues [121]. The activation of the MAPK pathway is divided in various cascades, the main ones being the ERK1/ERK2, JNK/SAPK and p-38 MAPK [121, 122]. RAS activates RAF, subsequently leading to the ERK1/ERK2 cascade, which is stimulated mainly by mitogenic factors and is associated to cell division and survival. Chemotherapeutic compounds can also stimulate and increase ERK1/ERK2 phosphorylation allowing them to play a role of balance in apoptosis and cell survival [123, 124]. JNK and p-38 MAPK cascades are stimulated differently, *via* genotoxic stress including chemotherapeutics compounds, and play roles in cell growth arrest, inflammation and apoptosis [122, 123]. In ovarian cancer, decreased MAPK activity by the JNK and p-38 cascades has been associated with platinum-resistant cancer models [125, 126]. On the contrary, ERK1/ERK2 are associated to survival and cell growth and their inhibition by the protein MKP3 sensitized ovarian cancer cells to cisplatin [127]. MAPK and PI3K pathways are interconnected and can influence each other [128]. In fact, the chemoresistance associated with AKT2 in ovarian cancer is related to the inability of cisplatin to activate JNK and p-38 to induce apoptosis. Indeed, AKT2 is responsible for inhibiting ASK1 and its downstream targets including JNK and p-38 [105]. Concerning endometrial cancer, MAPK and chemoresistance, not much has been studied so far but we can hypothesize that the effect observed would be similar to those reported in ovarian cancer models.

#### HER family

Another family of oncogenes to consider in gynecological cancers is the epidermal growth factor receptors EGFR (HER-1) and ErbB2 (HER-2), which are known for being cell-surface receptors tyrosine kinases being structurally similar. Indeed, EGFR and ErbB2 are known for being overexpressed in advanced stages of both ovarian and endometrial cancers and being markers of poor prognosis [129–136]. Both EGFR and ErbB2 receptors can activate various signaling pathways, including both the PI3K and MAPK (*via* RAS-RAF oncogenes) which provide survival signaling, cell growth to tumors and contribute to the overall acquisition of chemoresistance in gynecological cancers. Interestingly, it has been demonstrated that EGFR and ErbB2 overexpression, in association with the activation of PI3K and MAPK signaling pathways, increase resistance to cisplatin and paclitaxel in gynecological cancers [137–142]. It is worth noting that the effect of EGFR and ErbB2 on chemoresistance and prognosis is controversial in the literature. Some studies indicate no association with these oncogenes, but it is overall still worth considering for cancer research.

#### Estrogen receptors

An important characteristic unique to gynecological cancers is the high presence of estrogen and its corresponding receptors (ERα/β), thus promoting cell proliferation and tumorigenesis [143]. Estrogen binds to its receptor, dimerize, allowing its translocation from the cytoplasm to the nucleus, then bind on ERE (DNA Estrogen Response Element) and act as a transcription factor [144, 145]. Estrogen can also act in a non-genomic manner by binding with estrogen receptors, located on the plasma membrane, which then interact with other receptors such as IGF-1R and ErbB2 [146, 147]. Estrogen can also directly bind on a G-coupled protein, the GPR30 receptor, independently of the estrogen receptors [147]. These non-genomic interactions of estrogen induce activity for both the PI3K and MAPK pathways, pathways involved in the chemoresistance of gynecological cancer [146, 147]. It appears important to note that estrogen is strongly associated with chemoresistance mechanisms in both endometrial and ovarian cancers [148–150]. It has been demonstrated in endometrial cancer cells that estrogen can positively activate GRP78, thus preventing apoptosis and providing chemoresistance to both paclitaxel and cisplatin [149]. Another study demonstrated that estrogen can provide chemoresistance to paclitaxel treatment in ovarian cancer cells *via* the phosphorylation of AKT-ASK1 complex [150]. The use of hormonal therapy is much more prevalent in the context of breast cancer; consequently, more data is available with this model. Results show that higher levels of ERα in breast cancer are correlated with a chemoresistance status against cisplatin, paclitaxel and doxorubicin [151].

### Tumor suppressors

Up to now, survival pathways were discussed in relation to chemoresistance of gynecological cancers, however, tumor suppressors also play an important role in these mechanisms.

#### P53

A largely studied tumor suppressor in all cancers is P53. Briefly, P53 is a tumor suppressor protein who has various roles of protection against cancer including DNA repair, cell growth arrest and apoptosis which gave this protein the nickname ‘’guardian of the genome”. P53 a tetramer tightly regulated by MDM2 and can be stabilized/activated upon diverse stimuli including oncogene activation, DNA damage, starvation or hypoxia. P53 can act as a transcription factor and is involved in the regulation of many genes from different mechanisms to keep the cell in good condition [152, 153]. Nonetheless, P53 is highly mutated in gynecological cancers ( > 90% in ovaries and 25-85% in the endometrium) being an important factor for tumorigenesis and cancer initiation (Figure 2C) [92, 152, 154]. Also noteworthy, ovarian and endometrial cancers frequently overexpress P53, WT or mutant [154–156]. Considering the importance of P53 apoptosis pathway, it appears clear that this protein is involved in gynecological cancers response to chemotherapy. As previously stated, P53 is involved in the chemoresistance associated to the PI3K pathway alteration in gynecological cancers [99, 101, 102, 118–120]. Epithelial-mesenchymal transition (EMT), a process of tumor invasion and metastasis, is related to the inhibition of P53-dependent apoptosis mechanisms and involved in the resistance of ovarian cancer to paclitaxel and radiation [157]. P53 alterations are also associated, *via* many different mechanisms, to the resistance of platinum compounds in ovarian [158–165] and in endometrial cancers [166, 167]. P53 inactivation is associated with an increase of the mitochondrial BCL-2, an anti-apoptotic protein, and it has been demonstrated that upregulation of BCL-2 was responsible for the acquired chemoresistance to platinum compounds in gynecological cancers [168, 169]. Overall, P53 is easily the most widely studied tumor suppressor in gynecological malignancies and its importance in these types of cancer is irrefutable.

#### Prostate apoptosis response-4 (Par-4)

Finally, an interesting tumor suppressor for therapies, and also related to chemoresistance, has recently taken interest in the scientific community and is named Prostate apoptosis response 4 (Par-4). Par-4 is a very interesting protein because of its unique ability to induce apoptosis in a cancer-selective manner [170, 171]. Indeed, this unique mechanism of selectivity has been demonstrated in various models and also seemed to be involved in chemoresistance (including Tamoxifen, taxane and platinum agents) and tumorigenesis mechanisms [172–176]. As previously described, gynecological tissues are known for being hormone-dependent and, interestingly, it has been demonstrated that estrogen can downregulate Par-4 and thus could be involved in chemoresistance-associated mechanisms [177, 178]. A study demonstrated that Par-4 increase the apoptotic response to paclitaxel treatment in ovarian cancer cells [179]. Our laboratory recently published a manuscript indicating that the cleaved form of Par-4 was highly reduced/absent in chemoresistant gynecological cancers indicating a potential venue for this protein to overcome this hurdle. This inhibition was post-translational and regulated by the PI3K and MAPK pathways, previously described as being involved in chemoresistance mechanisms [178]. Except these studies, the role of Par-4 on chemoresistance in gynecological cancer has received very little attention. We do believe that further studies of this promising tumor suppressor would be a very interesting avenue for gynecological cancer therapeutics.

A table summarizing the chemoresistance mechanisms discussed is available (Table 1).

**Table 1:** Chemoresistance mechanisms in ovarian and endometrial cancers

Mechanism	Tissue	Resistance	Comments	Reference
Efflux pumps				
↑ P-glycoprotein	Ovary and endometrium	Taxanes and doxorubicin		[34–37]
↑ ATP7A/ATP7B	Ovary and endometrium	Platinum		[38–42]
DNA Repair mechanisms				
↑ NER	Ovary	Platinum		[48–53]
↓ MMR	Ovary and endometrium	Platinum		[56–61, 64, 65]
↓ BRCA1	Ovary	Taxane	provides sensitivity to platinum compounds	[80–85]
↓ BRCA2	Ovary	-	provides sensitivity to platinum compounds	[85, 86]
Signaling pathways				
↑ PI3K	Ovary	Platinum		[94, 95]
↑ AKT	Ovary and endometrium	Platinum, taxanes and doxorubicin		[95–105, 120, 150]
↓ PTEN	Ovary and endometrium	Platinum		[91, 94, 106–110]
↑ XIAP	Ovary and endometrium	Platinum, taxanes and doxorubicin	XIAP induces chemoresistance against cisplatin (endometrial and ovarian), taxane (ovarian) and doxorubicin (endometrial)	[97, 100, 113–118]
↓ JNK	Ovary	Platinum		[105, 125]
↓ p-38	Ovary	Platinum		[105, 125, 126]
↑ ERK1/2	Ovary	Platinum		[127]
↑ EGFR	Ovary	Taxanes		[141]
↑ ErbB2	Ovary and endometrium	Platinum and taxanes		[137–140, 142]
↑ GRP78	Endometrium	Platinum and taxanes	Estrogen-regulated	[149]
↑ ASK1	Endometrium	Taxane	Estrogen-regulated	[150]
↑ ERα	Breast	Platinum, taxanes and doxorubicin		[151]
↓/mut P53	Ovary and endometrium	Platinum, taxanes and radiation	Resistance to radiation have been observed in ovarian cancer only	[99, 101, 102, 118–120, 157–167]
↑ BCL-2	Ovary and endometrium	Platinum		[168, 169]
↓ PAR-4	Ovary	Taxane		[179]

The table summarizes the mechanisms of chemoresistance observed in ovarian and endometrial cancers. The different column indicates the mechanism and its regulation, the tissues concerned, the drug resistance, additional comments as well as the bibliographical references.

## TARGETED THERAPIES TO OVERCOME CHEMORESISTANCE

**Figure 3:**
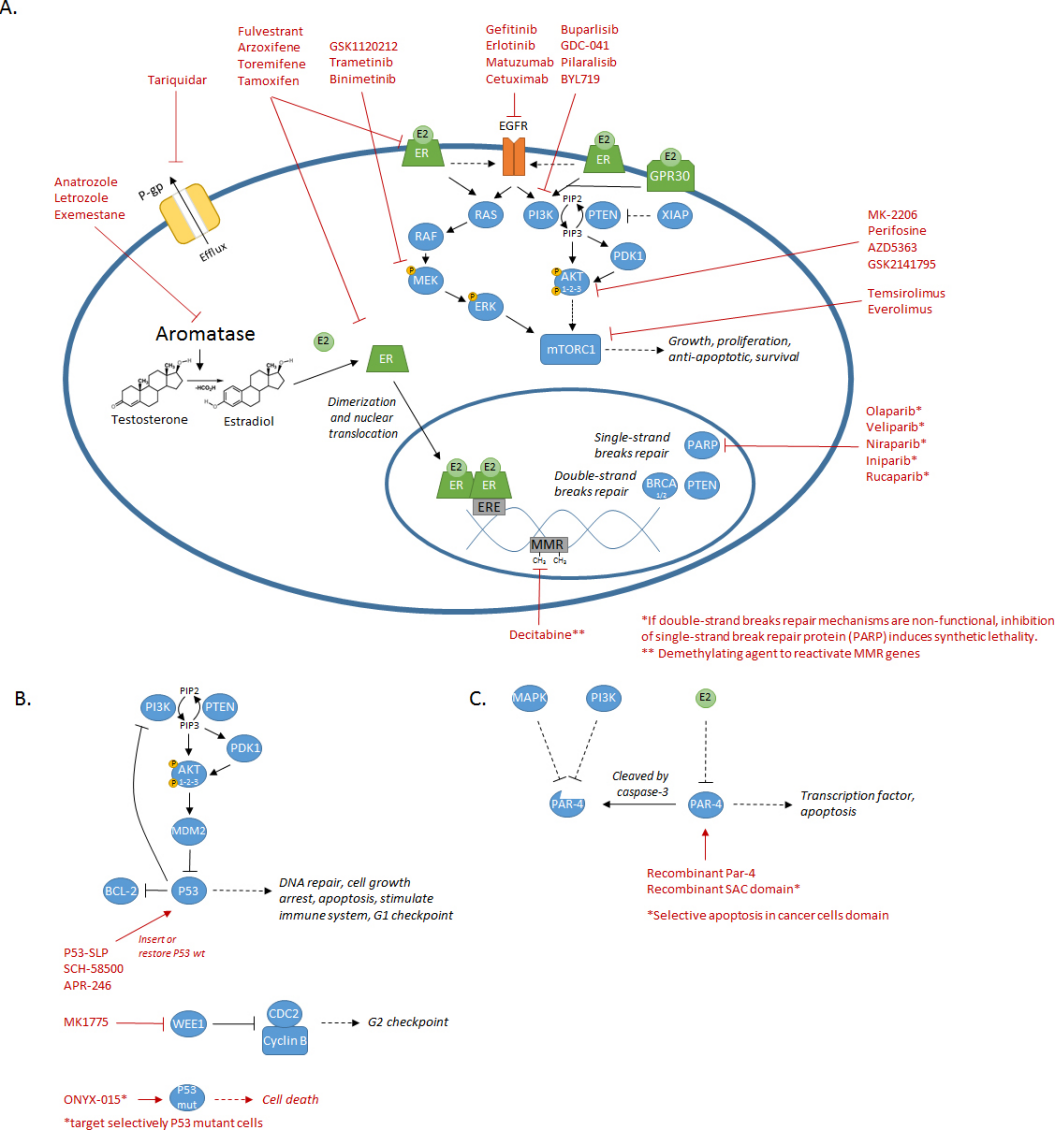
Summary of the diverse mechanisms/pathways involved in chemoresistance and their associated targeted treatments **A**. Schematic representing most of the mechanisms/pathways (P-gp, MMR, PARP, aromatase, ER, EGFR, PI3K/AKT, MAPK, mTOR) discussed and their associated therapeutics molecules. **B**. Schematic of the P53 mechanisms discussed and the diverse targeted therapies previously tested on ovarian and endometrial cancers. **C**. Schematic of the Par-4 mechanisms and the targeted therapy approach suggested using recombinant proteins (Par-4 or SAC domain only) on cancer cells.

Considering current therapies are not sufficient to overcome advanced gynecological cancers, new therapies are still being researched by the scientific community. Targeted therapy is a new approach of treatment that uses compounds which aim, in a specific manner, the cancer cells by attacking its oncogenic mechanisms. These mechanisms are also often linked with their chemoresistance status, so targeting these could sensitize cancer cells to standard chemotherapy. This section will discuss of the current targets and their associated drugs currently under study, specifically in gynecological cancers. A figure summarizing the previous mechanisms/pathways involved in chemoresistance as well as the molecules targeting these is available to ease the reading (Figure 3). A table is also available to summarize the different clinical trials discussed in this section (Supplementary Table 1).

### Targeting efflux pumps

The p-Glycoprotein, involved in chemoresistance mechanisms, is a target of interest in cancers and some drugs have been tested under clinical trials, specifically on gynecological tissues. First- (verapamil; cyclosporine) and second-generation (PSC833; VX-710) have been developed but the results in clinical trials were disappointing considering their low potency, the low specificity and the increase of toxicity when combined with other chemotherapeutic drugs such as paclitaxel in ovarian cancer patients [180–182].

Tariquidar (XR9576) is a third-generation Pgp inhibitor, showing increased specificity and potency to inhibit the drug efflux mediated by Pgp. In contrast to second-generation Pgp inhibitors, Tariquidar, combined with docetaxel, showed minimal toxicity, less systemic pharmacokinetic interaction and was well tolerated by patients (including ovarian and cervical cancers) [183]. Interestingly, both *in vitro* and *in vivo* experiments indicated that Tariquidar can completely reverse the resistance of ovarian cancer cells against doxorubicin and paclitaxel [184]. *Ex vivo* experiments using ovarian tumors biopsies also demonstrated that combination of Tariquidar with either doxorubicin or paclitaxel increased the efficiency of these standards chemotherapeutic treatments by decreasing the chemoresistance status [185]. Further studies in clinical trials are needed to determine if the effect observed on chemoresistance and increase of efficiency is reproducible the toxicity of the compound on various types of cancer.

### Targeting repair mechanisms

#### PARP inhibitors

As previously introduced, BRCA mutations are frequent and involved in the chemoresistance mechanisms of gynecological cancers. One idea of therapy that has been developed involves the protein poly-ADP ribose polymerase (PARP), responsible for DNA repair of single strand break. The principle of this therapy is to inhibit PARP and thus reduce the ability of the cell to repair single strand breaks; these accumulated breaks will eventually lead to double strand breaks during cell DNA replication. Normal cells will be able to repair these double strand breaks, in part through homologous recombination repair, however, BRCA-1/2 deficient cancer cells will not and thus will undergo chromosomal instability and apoptosis. This is because the BRCA-1/2 genes, when mutated, induces a defect in homologous recombination, and pairing this with PARP inhibition allow synthetic lethality of cancer cells [186]. BRCA-1/2 deficiency is not required when using PARP inhibitors but they will be a lot more efficient with patients bearing this mutation. Some inhibitors of PARP previously used in clinical trials for gynecological cancers are the following: Olaparib, Veliparib, Niraparib, Iniparib and Rucaparib.

Olaparib has been widely studied in ovarian cancer disease. Indeed, many phase II trials have demonstrated an important improvement, using this PARP inhibitor as monotherapy, on the progression-free survival (PFS) and good response rate for women with advanced ovarian cancer [187–190]. The first study tested Olaparib on 33 patients with advanced and recurrent ovarian cancer and 11 of these had an objective response rate (the sum of partial responses (PR) and complete responses (CR); ORR) [187]. The second study was large scale with 265 platinum-sensitive relapsed patients randomized in 2 groups (Olaparib *versus* placebo). The results obtained were significant with a median progression-free survival of 8.4 months *versus* 4.8 months for the Olaparib-treated *versus* placebo-treated patients respectively. Regardless of the BRCA status, patients treated with Olaparib had a decreased risk of progression on the long term [188]. Another trial tested Olaparib in 63 patients with advanced ovarian carcinoma. The findings were very interesting: 7 of the 17 (41%) patients with BCRA1/2 mutations had an ORR to Olaparib while 11 of the 46 (24%) patient without mutations also had an ORR [189]. These results demonstrated that PARP inhibition can also be efficient in absence of BRCA mutations and that other proteins might be involved in the homologous recombination of cancer cells. A phase II trial also tested Olaparib in 193 women with advanced platinum-resistant ovarian cancer and 60 obtained an ORR (31%) demonstrating again a clinical benefit similar to previous trials with chemoresistant patients [190]. Further studies tested Olaparib in combination with chemotherapy in ovarian cancers. A phase I/Ib study tested Olaparib in combination with carboplatin on 37 women with ovarian cancer and BRCA mutations. Their findings were 1 CR and 15 PR, but also found out that FOXO3a expression, a transcription factor negatively regulated by AKT and involved in the regulation of genes in favor of apoptosis, may be predictive of the response to the treatment [191, 192]. Another phase II trial tested Olaparib in combination with both paclitaxel and carboplatin in 162 women with recurrent and platinum-sensitive ovarian cancer. The progression-free survival was significantly improved in the combination group (12.2 months) *versus* the chemotherapy alone (9.6 months). Very interestingly, only 41 of 107 measurable patients had BRCA mutations, demonstrating again, a clinical benefit of PARP inhibitors on patients without BRCA mutations [193]. A phase II trial tested Olaparib in combination with cediranib, an anti-angiogenic agent, in 90 patients with recurrent platinum-sensitive ovarian cancer. Women treated with Olaparib alone had a median progressive-free survival of 9 months while those who received the combination had median of 17.7 months [194]. The combination of these drugs (Olaparib and cediranib) improved the survival of patients greatly and studies further testing this combination are currently going on.

The other clinical PARP inhibitors also underwent a limited number of clinical trials in ovarian cancer. Veliparib was tested in a phase II trial as a single agent in ovarian cancer patients with BRCA mutations either platinum-sensitive (20 patients) or platinum-resistant (30 patients). The ORR of all patients was of 26% (2 CR and 11 PR) and when compared with their platinum-sensitivity, the ORR was of 20% for the resistant group *versus* 35% for the sensitive group [195]. A second study also tested Veliparib in combination with cyclophosphamide, an alkylating agent, in a study group of 72 women with BRCA mutated ovarian cancer. No significant results were obtained from these experiments and the addition of Veliparib did not improve the response rate nor the PFS [196]. Another PARP inhibitor selective for PARP-1 and PARP-2, Niraparib, went under a phase I study (42 ovarian/peritoneal cancer patients) and the preliminaries antitumor results were 8 PR and 2 SD among the 20 BRCA mutations carrier patients while being 5 PR and 3 SD among the 22 WT BRCA patients. Platinum sensitivity of patients was also considered when analyzing the response rate. No significant difference was observed in the CBR from BRCA mutation carrier (50% for sensitive and 50% for resistant), however, CBR was twice lower in BRCA WT platinum-resistant patients (67% for sensitive *versus* 32% for resistant) [197]. Iniparib, an additional PARP inhibitor, had a very promising phase II trial involving 17 platinum-sensitive patients with recurrent ovarian cancer and was used in combination with carboplatin and gemcitabine, a nucleoside analog. The ORR of patients treated with carboplatin and gemcitabine alone is normally around 47% but adding Iniparib to the combination significantly increased the ORR to 71%. Additionally, the ORR did not seem to be associated with the BRCA status of the patients [198]. Iniparib, combined with paclitaxel and carboplatin, have also been tested in a group of 17 women with advanced or recurrent uterine carcinosarcoma (displaying histological features from both endometrium and the outer layer of the uterus). The results obtained were limited considering that only 4 patients responded to the treatment [199]. Still, PARP1 overexpression is present in uterine carcinomas and studies with PARP inhibitors should not be neglected because of the failure of Iniparib in those latter studies.

Finally, Rucaparib is another PARP1/2 inhibitor undergoing clinical studies in ovarian cancer. Rucaparib have been initially tested in a pre-clinical study with 39 ovarian cancer cell lines characterized for BCRA1/2, PTEN and their chemosensitivity to platinum compounds. Responses to platinum chemotherapy was associated with Rucaparib responses and combining the PARP inhibitor with topotecan, carboplatin, doxorubicin, paclitaxel or gemcitabine provided additive or synergistic effects resulting in increased apoptosis [200]. Phase I trial has been done using Rucaparib and 29 patients with advanced solid tumors including ovarian/peritoneal cancer (7 patients). Preliminary results were very interesting with 2 PR and 10 SD among the various doses of Rucaparib tested. The most interesting part of this trial is the efficiency observed in ovarian/peritoneal cancer patients, which account for half of the response rates obtained and a CBR of 86% (1 PR and 5 SD) [201]. A phase II trial has also been done testing Rucaparib in women with BRCA mutated advanced breast and ovarian cancer. 22 of the 27 patients tested for oral Rucaparib were ovarian cancer patients but the ORR with the different doses administered was only 15%. However, 12 of the 13 patients who received continuously Rucaparib achieved either CR, PR or SD for more than 12 weeks [202].

Even if BRCA mutations can also occur in endometrial cancers, they are sporadic and not much have been tested concerning these mutations through the use of PARP inhibitors. An aspect of endometrial cancers is their high mutation rates of PTEN (Figure 2B), which also has a phosphatase-independent role in genomic stability and homologous recombination [203]. Considering the repair role of PTEN for double strand break, Olaparib have been tested in endometrial cancers with this protein of interest (PTEN). Preclinical studies have been performed in endometrial cancer cells and one of these demonstrated, using 16 cell lines, that the PARP inhibitor Olaparib was efficient but not necessarily associated with the PTEN status of the cell line [204]. Another pre-clinical trial, however, demonstrated both *in vitro* and *in vivo* that Olaparib was efficient and the effect was related to the PTEN status of endometrial cancer cells [205]. A clinical case has also been done with Olaparib and a 58-year-old woman with metastatic and recurrent endometrial cancer. After treatment with the PARP inhibitor Olaparib, a significant reduction of brain metastases was observed as well as improvement of tumor-associated symptoms. This clinical case report, after a biopsy, also mention the absence of BRCA mutations but a loss of PTEN instead, thus indicating that PTEN status should be considered for administration of PARP inhibitors against gynecological cancers [206]. Considering these preliminary results, endometrial and PARP inhibitors should be further studied.

An important aspect of PARP inhibitors is their low toxicity for patients, a very desirable effect for cancer treatment and possible combination with other chemotherapeutic compounds. In addition, the platinum sensitivity of the patients was a good marker of efficiency when using different PARP inhibitors. Finally, patient who did not have BRCA mutations also had a clinical benefit, indicating that other biomarkers should be studied for PARP inhibitors efficiency, such as the protein PTEN which also has a role in genomic stability and homologous recombination. Further trials are thus required for measuring the efficiency of PARP inhibitors, which currently look like a very promising therapy to use in the context of advanced gynecological cancers.

#### Nucleotide excision repair (NER)

Nucleotide excision repair (NER) is also involved in the chemoresistance of gynecological cancer and therapies targeting this mechanism are also emerging. ERCC1 is a potential target involved in this repair mechanism and involved in platinum resistance. However no enzymatic activity related to this protein (ERCC1) is known, making it hard to target. Though, novel small molecules have been developed targeting the XPA-ERCC1 complex and thus reestablishing platinum sensitivity [207].

#### Mismatch repair (MMR)

Mismatch repair deficiency is known for being involved in cancer development as well as chemoresistance, and some research is currently going on to target this mechanism and provoke synthetic lethality, as previously discussed with PARP inhibitors and BRCA genes. The principle of synthetic lethality is based on the loss of two genes (one from the inhibitor and one from mutation) from the same pathway leading to cancer cell death while normal cells only lose one of these (from the inhibitor) and still survive. Potential targets have been identified to be targeted along with MMR deficiency: dihydrofolate reductase (DHFR), DNA polymerase β (POLβ), DNA polymerase γ (POLγ) and PTEN-induced putative kinase 1 (PINK1), all causing accumulation of oxidative DNA damage when inhibited and combined with MMR deficiency (MSH1-2-6) [208–210]. However, synthetic lethality has not yet been tested in patients with advanced ovarian tumors [211]. Hypermethylation of hMLH1 gene is in part responsible for the MMR deficiency observed in ovarian cancer. Using Decitabine, a demethylating agent, on chemoeresistant/MLH1 silenced ovarian cancer xenografts, an improvement of sensitivity to cisplatin, carboplatin, temozolomide (an alkylating agent), and epirubicin (an anthracycline drug) was observed. Additionally, Decitabine also permitted a re-expression of MLH1 in these ovarian cancer xenografts [212]. Phase I trial has been done with Decitabine combined with carboplatin on 10 patients with recurrent platinum-resistant ovarian cancers. Results were 1 CR and 3 SD for more than 6 months. Noteworthy, HOXA11 and BRCA1 cancer associated genes were demethylated after treatment [213]. The same group is pursuing in a phase II trial with 17 patients with recurrent platinum-resistant ovarian cancer. They found out that Decitabine, followed by carboplatin treatment, allowed efficient demethylation of RASSF1A, HOXA10, HOXA11 and MLH1, which correlated with the progression-free survival of the patients (median of 10.2 months). The response rate obtained was of 35% and half of the patients were free of progression after 6 months [214]. The use of demethylating agent should be considered as a possible treatment to overcome chemoresistance in gynecological cancers presenting MMR deficiency and hypermethylation of genes.

### Targeting PI3K/AKT and MAPK

Considering the PI3K/AKT pathway is one of the main pathways involved in tumorigenesis and being highly mutated in gynecological cancers, many drugs have been developed to target various proteins of this pathway and to increase the efficiency of anti-cancer treatment, partly *via* the loss of chemoresistance. First generation PI3K inhibitors, such as Wortmannin and LY294002, were developed and mainly used in promising pre-clinical studies to better understand this pathway and their implication in therapies against gynecological cancer cells. One of these pre-clinical studies demonstrated *in vivo*, using athymic mice bearing ovarian cancer cells, that administration of Wortmannin efficiently sensitized the cancer cells to cisplatin treatment [215]. Yet, because of their poor pharmacokinetics properties, a second generation of inhibitors have been developed being more selective and sometime isoform specific. Among these second-generation PI3K inhibitors, Buparlisib (BKM-120), GDC-0941 and Pilaralisib (XL-147) are pan-class I PI3K inhibitors, which have been tested in few trials for gynecological cancers.

#### PI3K inhibitors

A previous study involving Buparlisib, in combination with Olaparib (PARP inhibitor), administered *in vivo* in mice with BRCA-1 mutations demonstrated an important synergy, which leads to a phase I trial with 34 women bearing high-grade serous ovarian cancer or triple-negative breast cancer [216, 217]. Among the patients, 26 were known for having BRCA mutations, a biomarker of efficiency for PARP inhibitors. All dose combinations allowed the observation of clinical benefits among the patients being promising for further trials [217]. GDC-041 has been tested in two phases I trial in patients with advanced solid tumors [218, 219]. The first trial was tested with 49 patients and minimal clinical results were observed with only two patients with a PR; one of these was an endocervical tumor with mutations on PIK3CA. CA125 responses were also observed in three patients with ovarian cancer including one with known high PIK3CA gene copy number [218]. The second phase I trial with GDC-0941 had similar results. Among the 42 patients tested, clinical activity was observed only on two patients, including one with a PTEN negative ovarian cancer [219]. Pilaralisib has been used in a phase II study in 67 patients with advanced or recurrent endometrial carcinoma. The ORR was minimal and only two patients had CR and two other patients had PR. No association was made between the molecular alterations of the PI3K pathway (three of these patients had wild-type PTEN with PIK3R1 mutations, one had PTEN mutation) and the clinical activity observed [220]. BYL719 is a PI3KCA isoform specific inhibitor that has been tested in a phase I trial in 36 patients with diverse tumors with PIK3CA mutations. Results were 7 PR (including one from cervical, one from endometrium and one from the ovary) [221].

As introduced, PI3K inhibitors had a considerable success rate in preclinical studies, however, the clinical studies of PI3K inhibitors presented in gynecological cancer were not as successful. The PI3K network is vastly complex and ramified, including feedback loops, alternates signaling cascades and crosstalk, all plausibly explaining the observed ineffectiveness. Inhibition of the PI3K pathway is known for also activating the MAPK pathway *via* crosstalk and dual inhibition with MAPK inhibitors could be considerable. The opposite is also true: inhibiting the MAPK upregulates PI3K/AKT activity [178, 222, 223]. The PI3K inhibitor Buparlisib have been tested in combination with the MEK inhibitor Trametinib in a phase Ib trial with 113 patients with advanced solid tumors, including ovarian cancer. Results among the 21 patients with ovarian cancer were 1 CR, 5 PR and 10 SD for an ORR of 29%. Remarkably, Buparlisib was efficient almost exclusively in ovarian cancer, the exception being a PR observed in a KRAS mutated non-small cell lung Cancer patient. Also noteworthy was the fact that 19 ovarian cancer patients were KRAS mutated demonstrating an efficient way to overcome this aggressive mutation [224]. The PIK3CA isoform inhibitor, BYL719, has been tested in combination with the MEK inhibitor Binimetinib in 58 patients with advanced tumors and mutated with *RAS* or *BRAF.* Four patients with ovarian cancer had KRAS mutation and 3 of these had a PR with the combined treatment. A patient with endometrial cancer and a KRAS mutation also had a PR. Overall, among all patients, 5 had PR and 18 had SD indicating an interesting efficiency for advanced tumors, especially gynecological cancers [225]. This combination is of particular interest for patients with KRAS mutations considering this mutation is often associated with an absence of response when using PI3K/AKT/mTOR inhibitor alone in gynecological cancers [226].

The use of dual-inhibiting PI3K and mTOR is also considered because of the intricate relationship between these complexes. Considering this fact, NVP-BEZ235 has been developed as a molecule capable of inhibiting both PI3K and mTOR. Preclinical studies have been performed in both gynecological cancers using this new inhibitor. One of these demonstrated, *via* 18 ovarian cancer cell lines (cisplatin sensitive and resistant models), sensitization to cisplatin and a correlation of the effect observed with those bearing PI3K-activations mutations or PTEN deletions. They also used a transgenic murine model of ovarian cancer (LSL-*K-*rasG12D/+PtenloxP^/loxP^) treated with NVP-BEZ235 and observed a longer median of survival as well as apoptotic activity [227]. Another study made similar experiments, but this time using 13 endometrial cancer cell lines (possessing at least one alteration of *PTEN, PIK3CA* or *KRAS) and xenografts in nude mice in vivo. NVP-BEZ235 was efficient to eliminate cancer cells both in vitro and in vivo. Interestingly, cancer cells with PTEN mutations but without KRAS alterations demonstrated a higher sensitivity to NVP-BEZ235 than those with KRAS alterations. Considering these observations, they added a MAPK inhibitor,* PD98059 or U0126, and successfully sensitized the *K-RAS* mutants to NVP-BEZ235, again demonstrating the implication of MAPK in the efficiency of PI3K inhibitors [228].

#### AKT inhibitors

AKT is a downstream target of PI3K and an important kinase involved in many mechanisms. Considering PI3K is a kinase central to a large network, which entails all the previously stated problematic; targeting AKT, downstream of PI3K, is thus an interesting avenue. AKT specific inhibitors have been developed (MK-2206, Perifosine, AZD5363 and GSK2141795) and pre-clinical trials looked promising [229–235]. Following these, few trials have been done in gynecological cancer patients. MK-2206, an allosteric inhibitor of AKT, has been tested in a phase II trial in 36 women with recurrent endometrial cancer. PIK3CA status was checked on patients; 9 were mutated and 27 were wild-type. Each group only had one patient with a PR, indicating a limited activity of this AKT inhibitor administered as a single agent and independently of the PIK3CA status in endometrial cancer patients [236]. Phase I trial tested Perifosine, another AKT inhibitor, combined with docetaxel in 21 taxane-resistant ovarian cancer patients. One patient (PTEN mutant) had a PR, another patient (PIK3CA mutant) had a SD and two other patients (no PI3K mutations) also had a SD. Noteworthy, patients with KRAS mutations had a rapid progression indicating again a relation with this protein as an indicator of inefficiency for PI3K/AKT inhibitors. Again, the efficiency of this AKT inhibitor was limited and no direct correlation with mutations of the PI3K pathway was made [237]. AZD5363, a novel and potent AKT inhibitor, have been through a phase I trial with 92 patients bearing advanced tumors. Among these patients, only 2 obtained a PR (one endometrioid cancer of the ovary and one cervical cancer with either PIK3CA or AKT1 mutation) and one got a SD (endometrioid cancer of the ovary with PIK3CA mutations) [238]. The results obtained showed that efficiency was, again, minimal. GSK2141795, another AKT inhibitor, have been tested in a phase I trial in 12 patients with recurrent platinum-resistant ovarian cancer. 8 of the 12 patients had a SD while the 4 left had a progressive disease [239]. Another phase I trial tested GSK2141795, but with a large group of 66 patients with advanced tumors. Among these, 12 were endometrial cancer patients and only 2 had a SD (PIK3CA mutant and/or PTEN loss) [240]. Similarly to PI3K inhibitors, GSK2141795 has been tested in combination with GSK1120212, a MEK1/2 inhibitor, in 13 patients with diverse tumors. 3 of the 13 patients had weak tumor regression (2 patients with ovarian cancer and 1 with endometrial cancer). The results were limited here. The study was a dose-escalating trials, which could have an impact on the efficiency observed [241]. Overall, AKT inhibitors clinical activity was also limited when tested on human patients with gynecological cancers in clinical trials.

To better estimate the expected results following chemotherapy regimens and increase the low response rate previously observed when using PI3K/AKT/mTOR inhibitors, biomarkers are a very interesting option. A study including 140 patients (breast, cervical, endometrial, and ovarian cancers) from phase I program treated with PI3K/AKT/mTOR inhibitors, measured different potential biomarkers (PIK3CA, KRAS, NRAS, and BRAF) to see if a correlation was possible with the response rate of patients. PIK3CA mutations were detected in 25 patients and their response rate to PI3K/AKT/mTOR inhibitors was significantly higher (30%) when compared with patients without the mutation (10%) [242]. The PI3K network is large and additional combinations of inhibitors would be necessary to overcome the inefficiency observed. As suggested by previous studies, KRAS mutations seemed to be a good indicator of resistance to PI3K inhibitors and combination with MAPK inhibitors seemed to be one of the attractive avenues to overcome absence of response. Another interesting avenue could be the use of PARP inhibitors. PI3K inhibition has been associated with the loss of homologous recombination and the addition of a PARP inhibitor could provide synthetic lethality selectively to cancer cells [243]. Indeed, as previously stated, PTEN has a role in homologous recombination and its status is considerable for using PARP inhibitors [203]. All things considered, to overcome the limited benefit of PI3K/AKT/mTOR inhibitors, a better understanding of the patient’s mutation status combined with another inhibitor to prevent resistance to treatment would be an appealing avenue to increase the success rate of this targeted therapy.

### Targeting mTor

Mammalian target of rapamycin (mTOR) is the catalytic subunit of two distinct complexes, mTORC1 and mTORC2. Both complexes associate with different proteins, thus regulating different substrates and play crucially important roles in protein synthesis, growth and survival. Interestingly, mTORC1 is sensitive to rapamycin while mTORC2 is not. It should be noted that mTOR lies downstream of the PI3K-AKT signaling cascade, a pathway frequently mutated in gynecological cancers and of paramount importance in the control of cell fate [244]. As such, it is a prime target for drug targeting and requires, in our opinion, further investigations.

Temsirolimus, a water-soluble derivative of rapamycin, is a specific mTOR inhibitor which blocks protein synthesis related to survival and tumor growth of cancer cells. Temsirolimus only target mTOR activity in the mTORC1 complex. A phase II trial was done with temsirolimus on 54 patients with recurrent ovarian cancer. Results obtained were modest with 9 patients having a PR [245]. Another study tested temsirolimus on 5 chemoresistant patients with clear cell carcinoma of the ovary. Of the 5 patients, 1 had a PR and another 1 had a stable disease (SD) [246]. Temsirolimus have been tested in combination with bevacizumab, an anti-angiogenesis compound under trial, enrolled with 31 women with recurrent ovarian cancer (17 chemosensitives *versus* 14 chemoresistants). Of the 25 patients evaluable, 3 obtained a PR and 9 had a SD. Interestingly, the 3 PR were from the platinum-resistant group, a desirable effect on advanced and recurrent cancer therapy [247]. Overall, temsirolimus showed only a modest activity on chemoresistant women with ovarian cancer. Concerning endometrial cancer, clinical studies have also been performed. A phase II trial tested temsirolimus alone or in combination with hormonal therapies (a progestin and tamoxifen) on patients with advanced/recurrent endometrial cancer. This study was focused on the fact that the mTOR pathway could be involved in the resistance to hormonal therapy in endometrial cancers. Temsirolimus alone was tested with 50 patients divided in two groups, 29 with prior chemotherapy and 21 without prior chemotherapy. The response rate was similar between these two groups with an ORR of 22% (24% ORR prior chemotherapy; 19% ORR without prior chemotherapy). This study also concluded that combining megestrol acetate (a steroidal progestin), tamoxifen and temsirolimus did not improve the treatment efficiency and was associated with an increased toxicity [248]. Another phase II trial tested temsirolimus alone in 54 recurrent and metastatic endometrial cancer patients. This study also considered if the patient had previously received chemotherapy, which were distributed in two distinct groups. Of the 29 chemo-naive patients, 4 had a PR and 20 a SD. From the 25 chemo-treated patients, 1 had a PR and 12 a SD. The single agent activity was higher in chemo-naive patients and demonstrated a beneficial effect to arrest cancer progression in both groups. They also checked if PTEN status, related to the regulation of mTOR pathway, was associated with the response rate of temsirolimus and found no association. Therefore, they concluded temsirolimus response was PTEN-independent [249]. Two other phase II trials tested temsirolimus in combination with bevacizumab, an angiogenesis inhibitor, in recurrent endometrial cancers patients. One study had 26 patients and obtained 5 PR and 12 were progression free at 6 months [250]. The second study had 49 patients and obtained 1 CR, 11 PR and 23 were progression free at 6 months [251]. Overall, these studies demonstrated a certain efficiency against recurrent endometrial cancer but also a considerable toxicity when combining both these compounds.

Everolimus (RAD-001) is another mTOR inhibitor with a mechanism similar to rapamycin and selective to mTORC1. A pre-clinical study has been done using 58 transgenic mice with bilateral ovarian serous adenocarcinomas developed accompanied by ascites and peritoneal dissemination. Treating these mice with everolimus alone reduced tumor burden by 84% and ascites and peritoneal dissemination were detected in only 21% of the treated mice *versus* 74% in the placebo-treated animals [252]. They also tested everolimus alone and in combination with cisplatin against ovarian cancer both *in vitro* and *in vivo*. Using ovarian cancer cell lines, they found out that everolimus was efficient for inhibiting cell proliferation and when combined with cisplatin, enhanced apoptosis. They also observed similar findings *in vivo* using xenografts models (inhibition of tumor growth, decrease of ascites and increased treatment efficiency when combined with cisplatin). Noteworthy, everolimus was efficient only in cells with high AKT/mTOR activity [253]. These pre-clinical findings were promising for the treatment of ovarian cancers. In endometrial cancer, phase II trials have been done using everolimus. One of these, tested everolimus in patients with recurrent endometrial cancer that have previously received chemotherapy. After treatment, 6 of the 28 patients had a SD, thus a clinical benefit rate of 22% (CBR; sum of CR, PR and SD) [254]. They also performed another phase II trial testing everolimus in combination with letrozole, an aromatase inhibitor, with 35 patients with advanced endometrial cancer. Using hormonal therapy in combination with everolimus increased the CBR, as well as the addition of CR and PR to the results, to 40% indicating a high benefit, considerable for therapy [255]. Another phase II trial tested everolimus alone in 44 patients with advanced endometrial cancer (2/3 previously received chemotherapy). After 3 months, 36% had a non-progressive disease and after 6 months, 4 patients had a PR [256].

Considering the high level of PTEN mutations in endometrial cancer, many trials tested mTOR inhibitors in this tissue. Results obtained were overall interesting but the combination of an mTOR inhibitor with another drug/compound such as an aromatase inhibitor provided very interesting results and will be necessary for optimal therapies. It is also worth noting that these two compounds inhibiting mTOR (temsirolimus and everolimus) tested in clinics only inhibit the mTORC1 complex. The mTORC2 complex is involved in the phosphorylation of the AKT protein at S473 leading to a full activation of this important kinase from the PI3K/AKT pathway, playing an important role in survival and proliferation [257, 258]. Another important fact concerning mTORC2 complex is that it can also subsequently activate the MAPK survival pathway [258]. An alternative pathway, insensitive to classical mTORC1 inhibition, is thus present and requires further attention to increase the efficiency of treatments.

### Targeting EGFR

Considering the high level of mutation/overexpression of EGFR in gynecological cancers, some therapeutics compounds targeting this family of oncogene have been tested in gynecological cancers.

Low molecular weight tyrosine kinase inhibitors Gefitinib (ZD-1839) and Erlotinib (OSI-774) have been developed to inhibit EGFR activity and prevent tumor growth. Gefitinib alone was administered in a phase II trial in 26 women with advanced endometrial cancer and the results obtained were four patients with a PFS of more than 6 months, one had a CR and 7 had a SD [259]. In ovarian cancer, phase II trials also tested gefitinib as a single agent. The results indicated that the inhibitor was able to decrease EGFR and p-EGFR protein levels but had minimal clinical benefits observed when using the agent alone [260, 261]. A phase II study decided to assess Gefitinib effectiveness in combination with tamoxifen with 56 women with ovarian cancer resistant to platinum and taxane therapies. The results were not conclusive because of the inefficacy of the treatment against the resistant cancers; no additional side effects were observed when combining tamoxifen with Gefitinib [262]. A phase II trial assessed the efficacy of Gefitinib in combination with paclitaxel and carboplatin for both platinum-sensitive and platinum-resistant ovarian, tubal or peritoneal adenocarcinoma patients (total of 68 patients). The results obtained were interesting considering the ORR of 19.2% and 61.9% and the CBR was of 69.2% and 81.0% for resistant and sensitive groups respectively [263]. Phase I/II trial combined Gefitinib with oxaliplatin and vinorelbine (an inhibitor of microtubule assembly) in 33 women with advanced ovarian cancer were divided between platinum-sensitive and resistant groups. The ORR was of 23.8% (3 CR and 2 PR) and 90% (4 CR and 5 PR) in the resistant and sensitive groups respectively [264]. These results show that Gefitinib present high effectiveness only when combined with chemotherapeutic compounds, particularly in chemosensitive cancers.

A phase II trial tested Erlotinib, as a single agent, in 32 women affected by advanced endometrial cancer and the results obtained were 4 PR and 15 SD [265]. Another phase II trial tested Erlotinib as a single agent, but this time in 34 women attained with advanced ovarian cancer. The results were 2 PR and 14 SD, similar to the results observed with endometrial cancers [266]. A phase IB trial tested Erlotinib combined with both carboplatin and docetaxel in 23 women with chemo-naive ovarian cancer. Results obtained were 5 CR and 7 PR giving an ORR of 52% [267]. A phase II study combined Erlotinib with carboplatin for 50 women with advanced ovarian cancer. Results were an ORR 7% (1 PR) for the resistant group *versus* 57% (14 PR) for the sensitive group [268]. Again, this indicates a good clinical efficiency when combined with chemotherapy but most particularly for chemosensitive patients.

Monoclonal antibodies against EGFR have also been developed, Cetuximab (IMC-C225; Erbitux) and matuzumab (EMD-72000). A phase II trial tested matuzumab, as a single agent, in ovarian cancer for 37 patients who previously received platinum treatment and became chemoresistant, demonstrated that the treatment was well tolerated but only led to 7 SD indicating that the clinical activity was limited [269]. Similar results were observed in a phase II trial of cetuximab as monotherapy in ovarian cancer patients with recurrent diseases [270]. Cetuximab combined with carboplatin and paclitaxel have been tested in another phase II trial for 40 women as an initial therapy for advanced ovarian cancer. The therapy was well tolerated, however, the results obtained were not beneficial (no increase of the progression-free survival (PFS) of the patients) [271]. Another phase II trial tested cetuximab combined with carboplatin in 28 patients with platinum sensitive ovarian cancers. Following treatments, 9 patients had an ORR and 8 SD indicating a certain clinical activity of the monoclonal antibody while not being optimal for therapy [272]. The effect of cetuximab seems to be reproducible in endometrial cancer and a pre-clinical study demonstrated that the monoclonal antibody was able to inhibit cell growth and invasion in endometrial Hec-1a cells *in vitro* while being able to inhibit tumor growth, lymph nodes and lung metastasis *in vivo* [273].

Interestingly, these trials demonstrated that inhibiting EGFR in gynecological cancers is well tolerated but the efficiency is low when used alone, thus necessitating a combination with another chemotherapeutic drug. The combination of EGFR inhibitors with another drug was effective in chemosensitive patients while being less effective in chemoresistant patients.

### Targeting the estrogen signaling pathway

Two types of treatments have been developed to target the estrogen signaling pathway, aromatase inhibitors and estrogen receptor antagonists.

#### Aromatase inhibitors

Anastrozole is a potent non-steroidal aromatase inhibitor that had one phase II trial in 23 recurrent endometrial cancer patients. The results were minimal with only 2 PR and 2 SD [274]. A phase II trial was performed using Anastrazole as a single agent in 53 asymptomatic recurrent/persistent ovarian (43), peritoneal (7) and fallopian tube (3) cancer patients. Results were 1 patient with a PR and 68% of these with a SD (42% > 90 days; 15% > 180 days; 7% > 270 days; 4% > 360 days) [275]. The same research group also performed a phase II trial using both Anastrozole and EGFR inhibitor Gefitinib in 35 patients with asymptomatic recurrent/persistent ovarian (30), peritoneal (4) and fallopian tube (1) cancers. 23 patients were evaluable and results were 1 CR and 14 SD, thus having only modest activity [276].

Letrozole is another aromatase inhibitor under study. In a group of 28 patients with recurrent endometrial cancer, but never treated with chemotherapy previously, letrozole was studied as a single agent in a phase II trial. Results obtained were 1 CR, 2 PR and 11 SD indicating a modest antitumoral activity. Interestingly, different markers including the hormone receptors were screened but were not correlated with response to letrozole [277]. Another phase II study tested letrozole in combination with the clinical mTor inhibitor everolimus in 35 women with advanced recurrent endometrial cancer. The results were very interesting with 11 CR, 2 PR and 1 SD [255]. Their previous study using everolimus alone had a CBR of 21% and only SD [254]. The addition of letrozole increased the CBR to 40%, as well as the addition of CR and PR, indicating a benefit when adding this aromatase inhibitor [255]. Letrozole was also tested in 50 ovarian cancer patients in a phase II trial. No CR or PR was attained with tumors and only 10 patients had a SD. Noteworthy, they observed a correlation between the response to letrozole and high estrogen receptor level [278]. Another phase II study tested letrozole in 21 women with recurrent ovarian cancer. Following treatment, the results were 1 CR, 2 PR and 4 SD, again indicating a modest antitumoral activity. No association was found between hormone receptors and response to letrozole [279]. Another study preselected 33 patients with ovarian cancer, all with the presence of estrogen receptors, and tested letrozole in a phase II trial. Results obtained were 3 PR and 14 SD, indicating that a pre-selection of patients can increase the efficiency of letrozole a little to arrest the progression of the cancer [280]. Finally, a phase II trial tested letrozole, but this time with 31 patients with ovarian cancer both resistant to platinum and taxane therapies. Notably, all patients were positive for estrogen receptors, however, none had a CR, one had a PR and 7 had a SD. These results indicate that chemoresistance has an impact on aromatase inhibitors, especially letrozole here, and their efficiency to stabilize tumor progression [281].

Exemestane is a novel aromatase inhibitor and one trial tested this compound in refractory ovarian cancer patients who previously received platinum and taxane therapies. No CR or PR was obtained with the 24 patients, however, 8 had a SD > 14 weeks [282]. Noteworthy, exemestane had an important effect on the tumor progression as previously observed with other aromatase inhibitors.

#### Estrogen receptor antagonists

Fulvestrant (Faslodex), is a novel estrogen receptor (ER) antagonist providing increased proteasomal degradation of its target. A phase II trial tested this compound in advanced endometrial cancer with 31 patients (estrogen receptor positive) and 22 patients (estrogen receptor negative). No patients demonstrated a CR or PR in the absence of estrogen receptor while 1 CR and 4 PR was observed in patients expressing the estrogen receptor. The disease was stable in 4 patients in the ER-negative group *versus* 9 in the ER-positive one. Fulvestrant antitumoral activity was absent without the presence of ER and minimal when the target (ER) was present; however the antagonist was efficient for stabilizing tumor growth [283]. Concerning ovarian cancer, a phase II was performed using fulvestrant in 26 women with advanced disease. The results obtained were similar to the ones obtained with endometrial cancer, 1 CR, 1 PR and 9 SD indicating a weak antitumoral activity with great stabilization of tumor growth [284]. The same research group measured the estrogen receptor protein level with tissue microarray (TMA) and correlated the expression measured, positively, with the clinical benefit previously observed [285]. Overall, Fulvestrant is also efficient for stabilizing tumor growth but modest for decreasing tumor size when administered alone.

Arzoxifene is another estrogen receptor antagonist tested for both the mammary and uterine tissues. An interesting aspect of arzoxifene is the lack of uterotrophic effect, a considerable feature for its use in endometrial cancers as compared to tamoxifen. Indeed, phase II trials have been done in patients with advanced or recurrent endometrial cancers and the results were promising. Two of these trials were done with 100 patients from two multi-institutional studies and results obtained were a low toxicity with an ORR of 25% and 31 % respectively [286]. Another trial specifically selected 29 patients with advanced endometrial cancer expressing ER and/or progesterone receptor and not previously treated with chemotherapy. Results obtained were a low toxicity, 1 CR and 8 PR (ORR = 31%) with a median duration of response greater than 13 months [287].

Toremifene is an antiestrogen that had a study using ovarian cancer cell lines *in vitro* and patients with ovarian and uterine cancers *in vivo*. Results *in vitro* demonstrated that toremifene was able to increase significantly the efficiency of doxorubicin on ovarian cancer cell lines; some were initially resistant to doxorubicin. On the 8 patients tested, 3 had PR, 3 had SD but 2 also had a tumor progression [288]. This study is thus promising for the further clinical trials tested with gynecological cancers.

The minimal effect observed in these diverse trials can be partly due to the non-selectivity of patients considering the frequent loss of estrogen receptors in recurrent gynecological cancers. However, the antitumoral effect of these agents seemed minimal but they were able to efficiently stabilize the disease. A combination of a compound from this family targeting the estrogen signaling pathway with a chemotherapeutic drug could increase the efficiency of the treatment. A combination with two targeted pathways could also be an interesting avenue. This was demonstrated in the promising study combining Letrozole with an mTOR inhibitor, which provided astonishing results [289]. Our laboratory previously studied a new chemotherapeutic compound called VP-128, which contained both a steroid portion (E2) and a toxic portion (platinum). Preclinical studies shown that this combination targeting the estrogen receptor was promising and efficient in ovarian cancer. Both *in vitro* and *in vivo* studies demonstrated a significant antitumoral effect against selected tumors expressing the estrogen receptor alpha. The effect was only modest in tumors without the estrogen receptor, similarly to the clinical results observed in trials [290]. Considering these observations, we suggest that screening patients before using therapeutic compounds targeting the estrogen signaling pathway seems to be of paramount importance to insure the combination therapy potency against endometrial and ovarian cancers.

### Targeting P53

Another important tumor suppressor involved in chemoresistance mechanisms to consider is P53. Many therapeutic avenues have been studied concerning P53. Another aspect to consider is the high amount of gynecological cancers overexpressing P53, making this protein an interesting target for therapy [154–156]. However, because of its multiple mechanisms and mutations, high levels of effectiveness are hard to achieve. In gynecological cancers, few trials have been made to test the efficiency of P53 targeting, a protein highly altered in these cancers (Figure 2C).

Gene therapy has been tested with the P53 gene *via* diverse techniques to re-express the functional P53 protein and regain its functions to eliminate tumor cells. P53-SLP is a synthetic peptide-vaccine containing a peptide derived from the middle portion of the P53 protein. The goal of this vaccine is to stimulate the immune system, a known function of P53, to mount a cytotoxic response against tumor cells overexpressing P53. This vaccine (P53-SLP) was tested in a phase II trial with 20 patients with ovarian cancer. Results obtained were 2 SD and 18 patients had a progression of the tumor. Beside the weak clinical benefit, they confirmed that the vaccine was well tolerated and stimulated T-cell responses in patients which was the primary objective of this vaccine [291]. P53-SLP was also tested in a phase II trial with 10 patients with recurrent ovarian cancer, pre-treated with low-dose cyclophosphamide to improve the immunogenicity of the vaccine. They observed a higher number of IFN-γ-producing T cells when compared to their previous study testing P53-SLP alone. However, the clinical results were again minimal with 2 SD and 8 progressive disease [292]. They also tested P53-SLP on 20 patients with advanced ovarian cancer, which also had a secondary chemotherapy following vaccine treatment. The administration of P53-SLP before chemotherapy allowed 2 SD only. Following analyses indicated that the administration of P53-SLP did not enhance the efficiency of chemotherapy treatments, thus was not able to overcome the chemoresistance of advanced ovarian cancer patients [293]. SCH-58500 is another therapy consisting of a genetically engineered adenovirus, unable to replicate and containing the wild-type gene P53. Phase I/II trial have been performed using SCH-58500 combined with platinum-based therapy with 24 patients diagnosed with recurrent ovarian cancer. Results obtained were satisfying considering the success to efficiently re-express P53 in tumors and a decrease higher than 50% CA125 (8/16 of the evaluable patients), an ovarian tumor marker, observed when combining both SCH-58500 with platinum compounds [294]. On the long term, they also observed that patients who only received a single dose of SCH-58500 had a median survival of 5 months *versus* 13 months for those who received multiple doses of SCH-58500 [295]. Overall, the trial of SCH-58500 in combination with platinum compounds tested on recurrent ovarian cancers was promising and more successful than the use of peptides-based approach.

Another promising compound, ONYX-015, is an oncolytic adenovirus that replicates selectively in cancer cells with malfunctioning P53, followed by lysis to eliminate tumors. Indeed, the replication and cytopathogenicity of this adenovirus is blocked by WT-P53 and thus only replicate in mutant P53 tumors [296]. If P53 is responsible for the chemoresistance of cancer cells, these would be eliminated leaving sensitive cells only to be treated with standard chemotherapy. Phase I trial tested ONYX-015 as monotherapy on patients with recurrent ovarian cancer but observed no clinical effect when used alone [297]. No more trials of ONYX-015 have been performed in gynecological cancer but the compound is still promising and has been tested in combination with chemotherapy in other tissues (head, neck and gastrointestinal with metastases) giving excellent results with complete responses and being responsive against chemoresistant patients [298, 299].

MK-1775 is a small molecule inhibitor targeting WEE1, a kinase responsible for inactivating CDC2/Cyclin B complex, involved in the G2 checkpoint for DNA damage. Most cell type presenting mutant-P53 lack the G1 checkpoint for DNA damage. Thus, inactivating the second checkpoint (G2) with a WEE1 inhibitor can sensitize to chemotherapeutic treatments in gynecological cancers [300]. Some trials have been performed using this new inhibitor in combination with chemotherapy in ovarian cancer. Phase I trial combining MK-1775 with carboplatin and paclitaxel with 14 patients sensitive to platinum therapy obtained 11 PR and 3 SD; 7 of these were evaluable by CA125 with 3 CR and 4 PR [301]. A similar trial has been done in a phase II trial with MK-1775 combined with carboplatin and paclitaxel and was tested on 121 women with platinum sensitive ovarian cancer (59 received carboplatin/paclitaxel + MK-1775 while 62 only received carboplatin/paclitaxel). Progression-free survival was greater with the addition of MK-1775 when compared with carboplatin/paclitaxel only group. The overall response rate was of 81% for the group who received the combination of carboplatin/paclitaxel including MK-1775 *versus* an ORR of 74% for patients who received only carboplatin/paclitaxel, indicating an increase of efficiency for treatment *via* the inhibition of WEE1 [302]. Another phase II study tested MK-1775 in combination with carboplatin on 22 patients with recurrent and platinum resistant ovarian cancer. Following treatments, 6 patients had a PR and 9 had a SD. The progression-free survival median was 11 months. Considering that patients were platinum-resistant, results were still interesting [303]. A similar phase II study is currently undergoing and testing MK-1775 in combination with gemcitabine also in recurrent and platinum-resistant ovarian cancer patients [304]. MK-1775 is relatively new and seems promising for the treatment of P53-mutated ovarian cancers. Trials in endometrial cancers could also be of interest considering the high alteration rate of P53 in this tissue.

APR-246 is a small molecule with the ability to restore mutant P53 to its wild-type conformation, allowing the activation apoptotic mechanisms to eliminate cancer cells. Preclinical studies have demonstrated a high synergetic effect of the molecule in combination with platinum compounds in ovarian cancer models both *in vitro* and *in vivo*. These studies also demonstrated that the addition of APR-246 in combination with standard chemotherapy was able to sensitize ovarian cancer cells and overcome chemoresistance to cisplatin and doxorubicin [305, 306]. A Phase I/II trial is currently undergoing in 160 ovarian cancer patients treated with APR-246 combined with carboplatin and doxorubicin and preliminary results still show positive and similar effects to those observed in pre-clinical trials [307]. In general, P53 targeted therapy is still actively studied and impressive with the diversity of bioengineered compounds targeting this protein. The number of clinical trials studying therapies targeting P53 is limited in gynecological cancers but look promising.

### Targeting Par-4

Par-4 is a promising candidate for future clinical trials because of its unique ability to induce apoptosis only and selectively in cancer cells. Using tumor suppressors is an alternative for cancer treatment *via* the use of recombinant proteins or gene therapy to express the gene as previously done with P53. Concerning Par-4, very little experimental data is available concerning therapeutic applications. However, a few *in vivo* experiments are convincing for future clinical studies and development of treatments. A study has demonstrated that the delivery of Par-4 plasmid *via* nanoliposomes to tumors in nude mice increased the efficiency of Fluorouracil, a thymidylate synthase inhibitor, in cancer [308]. Considering that Par-4 can activate apoptotic mechanisms *via* paracrine signaling, recombinant Par-4 has been studied. The effects observed using a recombinant variant of either Par-4 or its SAC (Selective for Apoptosis in Cancer cells) domain was successful in inducing apoptosis in cancer cells *in vitro* [309]. Another team produced recombinant SAC domain of Par-4 derived from plants, in the optic of large-scale production, which also kept its pro-apoptotic capabilities [310]. Considering the SAC domain is sufficient for apoptosis and selective for cancer cells, future compounds or treatments could be designed based on its structure for targeted therapy.

**Figure 4:**
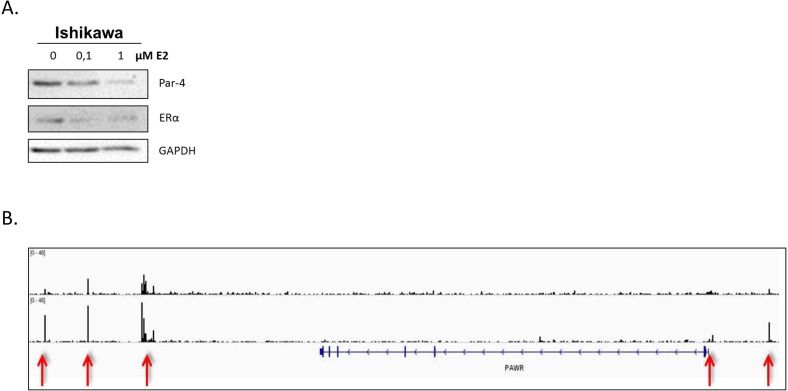
Estrogen implication in Par-4 regulation **A**. Ishikawa cancer cells were treated with either 0,1μM or 1μM estradiol (E2) for 4h. The levels of Par-4 and ERα were determined in treated cells using western blot analysis. GAPDH was used as a loading control. Results shown are representative of three independent experiments. **B**. ChIP-seq tracks showing ERα binding location on PAWR gene (Par-4) in control cells (top lane) and E2 treated cells (bottom lane) for 30 minutes. Genomic locations were obtained using the integrated genomic viewer (IGV 2.0). Red arrows indicate a novel ERα binding enrichment profile in the proximal region of Par-4 gene that can potentially be involved in negative regulation of the gene. Geo accession number: GSE23893.

Another option to consider concerning Par-4 targeted therapy is to combine with hormonal therapy. In prostate cancer, a study showed that Par-4 was efficient for inducing apoptosis only in hormone-independent cancer cells, indicating a role for hormone and Par-4 negative regulation [170, 311]. A study, involving 126 patients treated with neoadjuvant chemotherapy, assessed the expression of different genes to observe correlations between these and the outcome of the treatment. Results obtained were very interesting. Par-4 mRNA was upregulated following chemotherapy and Par-4 had a significant impact on prognostic, dependent of the ER status. Indeed, Par-4 level was predictive of a good outcome in ER- patients and the opposite was observed in ER+ patients indicating an important role for hormones and Par-4 [312]. As introduced, few studies demonstrated that estrogen can downregulate Par-4 [177, 178]. To further explore this mechanism of regulation, we used Gene Expression Omnibus (GEO) database and found a study (GSE23893) in uterine cancer tissues showing that ERα binds near Par-4 promoter in its proximal region indicating a potential link with estrogen regulation (Figure 4B). Considering the negative regulation observed with estrogen, we did a chromatin immunoprecipitation assay and found out that ERα binds near Par-4 promoter in its proximal region indicating a potential link with estrogen regulation (Figure 4B). Considering that the ovaries and endometrium are continuously under the influence of hormones and that estrogen exerts such a strong influence on Par-4 transcription, we are allowed to hypothesize that estrogen might play a role in the regulation of Par-4 expression. It is also possible that estrogen regulates Par-4 activity and localization, either through genomic or non-genomic mechanisms, further controlling Par-4 dynamics; estrogen could thus act as a potent carcinogenic agent as well as an inducer of chemoresistance depending on the situation. Combining hormonal therapy with Par-4 is thus a considerable option to acquire an optimal efficiency to induce apoptosis and reduce estrogen-driven growth stimulation. These preliminary findings concerning the estrogen regulation of Par-4 combined with its unique ability to selectively induce apoptosis in cancer cells only are very interesting and should be considered for future studies.

## FUTURE DIRECTIONS OF TARGETED THERAPIES

In light of the previous and current clinical trials reported in this review, our initial and primary observation is the modest amount of scrutiny endometrial cancer has been the subject of. Indeed, very few clinical studies have focused on endometrial cancer, probably because of its high survival rate in the case of early diagnosis. However, as introduced, endometrial cancer is very aggressive at advanced stage and the success rate of current therapies is very low. It is our opinion that, based on that fact, more investigations is required to develop novel therapies to improve the prognostic of women afflicted with recurrent endometrial cancer.

The idea of targeted-therapy is to eliminate gynecological cancer cells more selectively and turning-off their chemoresistance mechanisms. However, up to now, no cancer trials have been able to have the desired ‘’perfect” response rate *via* targeted therapy. Neither mono targeted-therapy nor combination with current chemotherapy regimen is sufficient to enhance greatly the survival of patients. As already observed under trials, combination of diverse targeted-therapies is an interesting avenue to overcome the resistance/absence of response seen with mono targeted-therapy. Indeed, many of the pathways and mechanisms discussed can have crosstalk with other pathways, feedback loop regulations in their large network or simply alternative regulation not inhibited by the compounds. For example, MAPK inhibition combined with PI3K inhibition increase significantly the efficiency of these compounds against gynecological cancers. Considering this fact, the most promising trials were those combining two targets such as Everolimus (targeting mTORC1) combined with Letrozole (targeting aromatase) or Buparlisib/BYL719 (compounds targeting PI3K) combined with Trametinib/binimetinib (compounds targeting MEK) [224, 225, 255].

Another avenue to consider for increasing the efficiency of targeted therapies would be to find specific biomarkers correlating with the response to treatment. Indeed, many of these new compounds have not yet, found efficient biomarkers to estimate the success of the treatment, which can also explain the modest clinical activity observed in many of these trials. Using biomarkers would lead to personalized medicine with a large pre-screening of the patients and the diverse mutations located in their tumors, which can be costly for phases II and III trials. This pre-screening, however, would allow to better identify the right combination of targeting compounds and obtain an optimal success rate of treatment.

The heterogeneity between and within tumors also plays a large role in the response rate of targeted treatments and chemoresistance mechanisms. Genomic instability is an important factor related to the heterogeneity of the tumor and, as stated in this review, repair mechanisms are involved in this instability as well as the PI3K network, which is vastly mutated in gynecological cancers [313]. This heterogeneity is reflected on the recurrence of gynecological cancers at advanced stages where only sensitive cells are dying to leave a resistant population, which will eventually come back. Heterogeneity of the tumors is also related to cancer stem cells (CSC). CSC are a small population of the tumor which have the capacities to initiate tumor, self-renew and differentiate to make the bulk of the tumor. They also have the ability to metastasize, an event occurring in advanced stages of cancer. One characteristic of these CSC is that they also play a role in the resurgence of the tumor following chemotherapy, indicating that they also resist chemotherapeutic treatments which is likely associated with a poor prognosis [314, 315]. The presence of chemoresistant and tumorigenic CSC has already been observed in ovarian cancer, however, these are still difficult to identify and target [316–319]. Noteworthy, one of these mechanisms of resistance is also related to AKT, a protein implicated in the PI3K network which is highly mutated in ovarian and endometrial cancers [320]. The biology of CSC is unique and their mechanisms of resistance are diverse. Many trials targeting CSC are currently under study. Considering their important role in tumor, this particular type of cell should be considered when targeting tumors to prevent recurrence and improve success rate.

Overall, considering that heterogeneity is a major hurdle for chemotherapy, targeting multiple pathways/proteins would be useful to overcome a maximum of cancer cells, including stem cells, during treatment. A better understanding of patients’ parameters will be profitable for the use of targeted-therapies.

Other elements to consider for efficient targeted-therapy against chemoresistant cancer cells are the fact that not only the modifications occurring inside the cells contribute to the chemoresistance. This manuscript mainly focus on this aspect, however, tumor cell micro-environment and the pharmacokinetic of compounds also play important roles on the acquisition of chemoresistance and should be considered for future therapies [13]. Concerning microenvironment, hypoxia is known for being related to radioresistance and chemoresistance of cancer [321, 322]. Hypoxic cancer cells, frequently located in the center of the solid tumor, have fewer blood vessels and consequently are less exposed to cancer drugs. Hypoxia is also involved in a slower proliferation rate, which affect current chemotherapy targeting cells with rapid division [322]. This microenvironment factor is thus a negative key player for the success of cancer therapies. Noteworthy, cancer stem cells also take advantage of the tumor microenvironment [314]. The immune system also interacts with the microenvironment of tumors as well as the extracellular matrix and signaling molecules from the environment. These microenvironment factors are considered in clinic and therapies targeting angiogenesis, hypoxia, immune system or tyrosine kinase receptors are currently under study [323]. A combination of treatment including these could be positive for the success rate of cancer treatment with other targeted-therapies.

Cancer is a complex disease and still requires research and investigations to better understand it. Current results indicate that mono-targeted therapies are not enough to overcome tumor progression and its resistance to various treatments. However, therapies and molecules are improving and the advance in technology allows a more precise diagnosis of the patients. A better understanding of tumor genetics will allow the administration of an efficient personalized medicine in gynecological cancers.

## SUPPLEMENTARY TABLE



## Abbreviations

CR: Complete response
PR: Partial response (at least 30% reduction of the tumor)
SD: Stable disease (between 30% reduction and 25% increase of the tumor)
PFS: Progression Free Survival
ORR: Objective response rate (CR+PR)
CBR: Clinical benefit rate (CR+PR+SD)
ER: Estrogen receptor
LHRH: Luteinizing hormone-releasing hormone
NER: Nucleotide excision repair
MMR: Mismatch repair
ERE: Estrogen response element
EMT: Epithelial-mesenchymal transition
Pgp: p-Glycoprotein
PARP: Poly-ADP ribose polymerase
DHFR: Dihydrofolate reductase
POLβ: DNA polymerase β
POLγ: DNA polymerase γ
PINK1: PTEN-induced putative kinase 1
TMA: Tissue microarray
E2: Estradiol
CSC: Cancer stem cell

## References

[1] Ferlay J, Steliarova-Foucher E, Lortet-Tieulent J, Rosso S, Coebergh JWW, Comber H, Forman D, Bray F (2013). Cancer incidence and mortality patterns in Europe: Estimates for 40 countries in 2012. European Journal of Cancer.

[2] American Cancer Society (2015). Cancer Facts & Figures.

[3] Canadian Cancer (2015). Society’s Advisory Committee on Cancer Statistics. Canadian Cancer Statistics.

[4] Siegel R, Naishadham D, Jemal A (2013). Cancer statistics, 2013. CA Cancer J Clin.

[5] Casciato DA, Territo MC (2009). Manual of clinical oncology.

[6] Howlader N, Noone A, Krapcho M, Miller D, Bishop K, Altekruse S, Kosary C, Yu M, Ruhl J, Tatalovich Z, Mariotto A, Lewis D, Chen H (2016). SEER Cancer Statistics Review, 1975-2013. National Cancer Institute.

[7] Lengyel E Ovarian Cancer Development and Metastasis. The American Journal of Pathology.

[8] Foley OW, Del Carmen MG (2013). Recurrent epithelial ovarian cancer: an update on treatment. Oncology.

[9] Smith RA, von Eschenbach AC, Wender R, Levin B, Byers T, Rothenberger D, Brooks D, Creasman W, Cohen C, Runowicz C (2001). American Cancer Society guidelines for the early detection of cancer: update of early detection guidelines for prostate, colorectal, and endometrial cancers: Also: update 2001—testing for early lung cancer detection. CA Cancer J Clin.

[10] Plataniotis G, Castiglione M, Group EGW (2010). Endometrial cancer: ESMO Clinical Practice Guidelines for diagnosis, treatment and follow-up. Annals of Oncology.

[11] Patch A-M, Christie EL, Etemadmoghadam D, Garsed DW, George J, Fereday S, Nones K, Cowin P, Alsop K, Bailey PJ (2015). Whole–genome characterization of chemoresistant ovarian cancer. Nature.

[12] Bast RC, Hennessy B, Mills GB (2009). The biology of ovarian cancer: new opportunities for translation. Nature Reviews Cancer.

[13] Agarwal R, Kaye SB (2003). Ovarian cancer: strategies for overcoming resistance to chemotherapy. Nature Reviews Cancer.

[14] American Cancer Society (2016). Endometrial (Uterine) cancer detailed guide.

[15] American Cancer Society (2016). Ovarian cancer detailed guide.

[16] Sjoquist KM, Martyn J, Edmondson RJ, Friedlander ML (2011). The role of hormonal therapy in gynecological cancers-current status and future directions. International Journal of Gynecological Cancer.

[17] Garrett A, Quinn MA (2008). Hormonal therapies and gynaecological cancers. Best Practice & Research Clinical Obstetrics & Gynaecology.

[18] Kreizman-Shefer H, Pricop J, Goldman S, Elmalah I, Shalev E (2014). Distribution of estrogen and progesterone receptors isoforms in endometrial cancer. Diagnostic Pathology.

[19] Issa RM, Lebeau A, Grob T, Holst F, Moch H, Terracciano L, Choschzick M, Sauter G, Simon R (2008). Estrogen receptor gene amplification occurs rarely in ovarian cancer. Mod Pathol.

[20] Cunat S, Hoffmann P, Pujol P (2004). Estrogens and epithelial ovarian cancer. Gynecologic Oncology.

[21] Pujol P, Rey JM, Nirde P, Roger P, Gastaldi M, Laffargue F, Rochefort H, Maudelonde T (1998). Differential expression of estrogen receptor-alpha and -beta messenger RNAs as a potential marker of ovarian carcinogenesis. Cancer Res.

[22] Kim JJ, Chapman-Davis E (2010). Role of Progesterone in Endometrial Cancer. Semin Reprod Med.

[23] Song JY, Fraser I (1995). Effects of progestogens on human endometrium. Obstetrical & gynecological survey.

[24] Gould RE, Garcia AA (2006). Update on aromatase inhibitors in breast cancer. Current Opinion in Obstetrics and Gynecology.

[25] Lalwani N, Prasad SR, Vikram R, Shanbhogue AK, Huettner PC, Fasih N (2011). Histologic, molecular, and cytogenetic features of ovarian cancers: implications for diagnosis and treatment. Radiographics.

[26] Wang D, Lippard SJ (2005). Cellular processing of platinum anticancer drugs. Nat Rev Drug Discov.

[27] Orr GA, Verdier-Pinard P, McDaid H, Horwitz SB (2003). Mechanisms of Taxol resistance related to microtubules. Oncogene.

[28] Jordan MA, Wilson L (2004). Microtubules as a target for anticancer drugs. Nat Rev Cancer.

[29] Thorn CF, Oshiro C, Marsh S, Hernandez-Boussard T, McLeod H, Klein TE, Altman RB (2011). Doxorubicin pathways: pharmacodynamics and adverse effects. Pharmacogenetics and genomics.

[30] Boere IA, E L, van der Burg M (2012). Review of dose-intense platinum and/or paclitaxel containing chemotherapy in advanced and recurrent epithelial ovarian cancer. Current pharmaceutical design.

[31] Yap TA, Carden CP, Kaye SB (2009). Beyond chemotherapy: targeted therapies in ovarian cancer. Nat Rev Cancer.

[32] Moxley KM, McMeekin DS (2010). Endometrial Carcinoma: A Review of Chemotherapy, Drug Resistance, and the Search for New Agents. The Oncologist.

[33] Longley D, Johnston P (2005). Molecular mechanisms of drug resistance. The Journal of pathology.

[34] Esteller M, Martinez‐Palones JM, García A, Xercavins J, Reventós J (1995). High rate of MDR‐1 and heterogeneous pattern of MRP expression without gene amplification in endometrial cancer. International journal of cancer.

[35] Schneider J, Centeno M, Rodriguez-Escudero F, Efferth T, Mattern J, Volm M (1993). High rate of expression of multidrug resistance-associated P-glycoprotein in human endometrial carcinoma and normal endometrial tissue. European Journal of Cancer.

[36] Holzmayer TA, Hilsenbeck S, Von Hoff DD, Roninson IB (1992). Clinical Correlates of MDR1 (P-glycoprotein) Gene Expression in Ovarian and Small-Cell Lung Carcinomas. Journal of the National Cancer Institute.

[37] Kamazawa S, Kigawa J, Kanamori Y, Itamochi H, Sato S, Iba T, Terakawa N (2002). Multidrug Resistance Gene-1 Is a Useful Predictor of Paclitaxel-Based Chemotherapy for Patients with Ovarian Cancer. Gynecologic Oncology.

[38] Katano K, Kondo A, Safaei R, Holzer A, Samimi G, Mishima M, Kuo Y-M, Rochdi M, Howell SB (2002). Acquisition of Resistance to Cisplatin Is Accompanied by Changes in the Cellular Pharmacology of Copper. Cancer Research.

[39] Nakayama K, Kanzaki A, Ogawa K, Miyazaki K, Neamati N, Takebayashi Y (2002). Copper-transporting P-type adenosine triphosphatase (ATP7B) as a cisplatin based chemoresistance marker in ovarian carcinoma: Comparative analysis with expression of MDR1, MRP1, MRP2, LRP and BCRP. International Journal of Cancer.

[40] Aida T, Takebayashi Y, Shimizu T, Okamura C, Higasimoto M, Kanzaki A, Nakayama K, Terada K, Sugiyama T, Miyazaki K, Ito K, Takenoshita S, Yaegashi N (2004). Expression of copper-transporting P-type adenosine triphosphatase (ATP7B) as a prognostic factor in human endometrial carcinoma. Gynecologic Oncology.

[41] Komatsu M, Sumizawa T, Mutoh M, Chen Z-S, Terada K, Furukawa T, Yang X-L, Gao H, Miura N, Sugiyama T, Akiyama S-i (2000). Copper-transporting P-Type Adenosine Triphosphatase (ATP7B) Is Associated with Cisplatin Resistance. Cancer Research.

[42] Samimi G, Safaei R, Katano K, Holzer AK, Rochdi M, Tomioka M, Goodman M, Howell SB (2004). Increased Expression of the Copper Efflux Transporter ATP7A Mediates Resistance to Cisplatin, Carboplatin, and Oxaliplatin in Ovarian Cancer Cells. Clinical Cancer Research.

[43] Xu W, Cai B, Chen JL, Li LX, Zhang JR, Sun YY, Wan XP (2008). ATP7B antisense oligodeoxynucleotides increase the cisplatin sensitivity of human ovarian cancer cell line SKOV3ipl. International Journal of Gynecological Cancer.

[44] Parker RJ, Eastman A, Bostick-Bruton F, Reed E (1991). Acquired cisplatin resistance in human ovarian cancer cells is associated with enhanced repair of cisplatin-DNA lesions and reduced drug accumulation. Journal of Clinical Investigation.

[45] Masuda H, Tanaka T, Matsuda H, Kusaba I (1990). Increased Removal of DNA-bound Platinum in a Human Ovarian Cancer Cell Line Resistant to cis-Diamminedichloroplatinum(II). Cancer Research.

[46] Johnson SW, Perez RP, Godwin AK, Yeung AT, Handel LM, Ozols RF, Hamilton TC (1994). Role of platinum-DNA adduct formation and removal in cisplatin resistance in human ovarian cancer cell lines. Biochemical Pharmacology.

[47] Lai G-M, Ozols RF, Smyth JF, Young RC, Hamilton TC (1988). Enhanced DNA repair and resistance to cisplatin in human ovarian cancer. Biochemical pharmacology.

[48] Selvakumaran M, Pisarcik DA, Bao R, Yeung AT, Hamilton TC (2003). Enhanced Cisplatin Cytotoxicity by Disturbing the Nucleotide Excision Repair Pathway in Ovarian Cancer Cell Lines. Cancer Research.

[49] Ferry KV, Hamilton TC, Johnson SW (2000). Increased nucleotide excision repair in cisplatin-resistant ovarian cancer cells: Role of ercc1–xpf. Biochemical Pharmacology.

[50] Dabholkar M, Vionnet J, Bostick-Bruton F, Yu JJ, Reed E (1994). Messenger RNA levels of XPAC and ERCC1 in ovarian cancer tissue correlate with response to platinum-based chemotherapy. Journal of Clinical Investigation.

[51] Reed E (1998). Platinum-DNA adduct, nucleotide excision repair and platinum based anti-cancer chemotherapy. Cancer treatment reviews.

[52] Chang I-Y, Kim M-H, Kim HB, Kim S-H, Kim H-Y, You HJ (2005). Small interfering RNA-induced suppression of ERCC1 enhances sensitivity of human cancer cells to cisplatin. Biochemical and biophysical research communications.

[53] Stevens EV, Nishizuka S, Antony S, Reimers M, Varma S, Young L, Munson PJ, Weinstein JN, Kohn EC, Pommier Y (2008). Predicting cisplatin and trabectedin drug sensitivity in ovarian and colon cancers. Molecular cancer therapeutics.

[54] Kunkel TA, Erie DA (2005). DNA mismatch repair*. Annu Rev Biochem.

[55] Martin LP, Hamilton TC, Schilder RJ (2008). Platinum Resistance: The Role of DNA Repair Pathways. Clinical Cancer Research.

[56] Samimi G, Fink D, Varki NM, Husain A, Hoskins WJ, Alberts DS, Howell SB (2000). Analysis of MLH1 and MSH2 expression in ovarian cancer before and after platinum drug-based chemotherapy. Clinical cancer research.

[57] Drummond JT, Anthoney A, Brown R, Modrich P (1996). Cisplatin and adriamycin resistance are associated with MutLα and mismatch repair deficiency in an ovarian tumor cell line. Journal of Biological Chemistry.

[58] Strathdee G, MacKean M, Illand M, Brown R (1999). A role for methylation of the hMLH1 promoter in loss of hMLH1 expression and drug resistance in ovarian cancer. Oncogene.

[59] Brown R, Hirst GL, Gallagher WM, McIlwrath AJ, Margison GP, van der Zee AG, Anthoney DA (1997). hMLH1 expression and cellular responses of ovarian tumour cells to treatment with cytotoxic anticancer agents. Oncogene.

[60] Aebi S, Kurdi-Haidar B, Gordon R, Cenni B, Zheng H, Fink D, Christen RD, Boland CR, Koi M, Fishel R (1996). Loss of DNA mismatch repair in acquired resistance to cisplatin. Cancer research.

[61] Zeller C, Dai W, Steele NL, Siddiq A, Walley AJ, Wilhelm-Benartzi C, Rizzo S, van der Zee A, Plumb J, Brown R (2012). Candidate DNA methylation drivers of acquired cisplatin resistance in ovarian cancer identified by methylome and expression profiling. Oncogene.

[62] Helleman J, Staveren IL, Dinjens WN, Kuijk PF, Ritstier K, Ewing PC, Burg ME, Stoter G, Berns EM (2006). Mismatch repair and treatment resistance in ovarian cancer. BMC cancer.

[63] Mesquita B, Veiga I, Pereira D, Tavares A, Pinto IM, Pinto C, Teixeira MR, Castedo S (2005). No significant role for beta tubulin mutations and mismatch repair defects in ovarian cancer resistance to paclitaxel/cisplatin. BMC cancer.

[64] Fink D, Nebel S, Aebi S, Zheng H, Cenni B, Nehmé A, Christen RD, Howell SB (1996). The Role of DNA Mismatch Repair in Platinum Drug Resistance. Cancer Research.

[65] Lin X, Howell SB (1999). Effect of loss of DNA mismatch repair on development of topotecan-, gemcitabine-, and paclitaxel-resistant variants after exposure to cisplatin. Molecular pharmacology.

[66] Esteller M, Catasus L, Matias-Guiu X, Mutter GL, Prat J, Baylin SB, Herman JG (1999). hMLH1 promoter hypermethylation is an early event in human endometrial tumorigenesis. The American journal of pathology.

[67] Kanaya T, Kyo S, Maida Y, Yatabe N, Tanaka M, Nakamura M, Inoue M (2003). Frequent hypermethylation of MLH1 promoter in normal endometrium of patients with endometrial cancers. Oncogene.

[68] Gurin CC, Federici MG, Kang L, Boyd J (1999). Causes and Consequences of Microsatellite Instability in Endometrial Carcinoma. Cancer Research.

[69] Masuda K, Banno K, Yanokura M, Kobayashi Y, Kisu I, Ueki A, Ono A, Asahara N, Nomura H, Hirasawa A, Susumu N, Aoki D (2011). Relationship between DNA Mismatch Repair Deficiency and Endometrial Cancer. Molecular Biology International.

[70] Welcsh PL, King M-C (2001). BRCA1 and BRCA2 and the genetics of breast and ovarian cancer. Human Molecular Genetics.

[71] Miki Y, Swensen J, Shattuck-Eidens D, Futreal PA, Harshman K, Tavtigian S, Liu Q, Cochran C, Bennett LM, Ding W (1994). A strong candidate for the breast and ovarian cancer susceptibility gene BRCA1. Science.

[72] Brose MS, Rebbeck TR, Calzone KA, Stopfer JE, Nathanson KL, Weber BL (2002). Cancer Risk Estimates for BRCA1 Mutation Carriers Identified in a Risk Evaluation Program. Journal of the National Cancer Institute.

[73] Petrucelli N, Daly MB, Feldman GL (2013). BRCA1 and BRCA2 hereditary breast and ovarian cancer.

[74] Pal T, Permuth-Wey J, Betts JA, Krischer JP, Fiorica J, Arango H, LaPolla J, Hoffman M, Martino MA, Wakeley K, Wilbanks G, Nicosia S, Cantor A (2005). BRCA1 and BRCA2 mutations account for a large proportion of ovarian carcinoma cases. Cancer.

[75] King M-C, Marks JH, Mandell JB, Group NYBCS (2003). Breast and ovarian cancer risks due to inherited mutations in BRCA1 and BRCA2. Science.

[76] Segev Y, Iqbal J, Lubinski J, Gronwald J, Lynch HT, Moller P, Ghadirian P, Rosen B, Tung N, Kim-Sing C (2013). The incidence of endometrial cancer in women with BRCA1 and BRCA2 mutations: an international prospective cohort study. Gynecologic oncology.

[77] Beiner ME, Finch A, Rosen B, Lubinski J, Moller P, Ghadirian P, Lynch HT, Friedman E, Sun P, Narod SA (2007). The risk of endometrial cancer in women with BRCA1 and BRCA2 mutations. A prospective study. Gynecologic oncology.

[78] Thompson D, Easton DF, Consortium BCL (2002). Cancer incidence in BRCA1 mutation carriers. Journal of the National Cancer Institute.

[79] Russell PA, Pharoah PD, De Foy K, Ramus SJ, Symmonds I, Wilson A, Scott I, Ponder BA, Gayther SA (2000). Frequent loss of BRCA1 mRNA and protein expression in sporadic ovarian cancers. International journal of cancer.

[80] Quinn JE, James CR, Stewart GE, Mulligan JM, White P, Chang GK, Mullan PB, Johnston PG, Wilson RH, Harkin DP (2007). BRCA1 mRNA expression levels predict for overall survival in ovarian cancer after chemotherapy. Clinical Cancer Research.

[81] Husain A, He G, Venkatraman ES, Spriggs DR (1998). BRCA1 up-regulation is associated with repair-mediated resistance to cis-diamminedichloroplatinum (II). Cancer research.

[82] Sylvain V, Lafarge S, Bignon Y-J (2002). Dominant-negative activity of a Brca1 truncation mutant: effects on proliferation, tumorigenicity in vivo, and chemosensitivity in a mouse ovarian cancer cell line. International journal of oncology.

[83] Xing D, Orsulic S (2006). A mouse model for the molecular characterization of brca1-associated ovarian carcinoma. Cancer research.

[84] Bartz SR, Zhang Z, Burchard J, Imakura M, Martin M, Palmieri A, Needham R, Guo J, Gordon M, Chung N (2006). Small interfering RNA screens reveal enhanced cisplatin cytotoxicity in tumor cells having both BRCA network and TP53 disruptions. Molecular and cellular biology.

[85] Tan DS, Rothermundt C, Thomas K, Bancroft E, Eeles R, Shanley S, Ardern-Jones A, Norman A, Kaye SB, Gore ME (2008). “BRCAness” syndrome in ovarian cancer: a case-control study describing the clinical features and outcome of patients with epithelial ovarian cancer associated with BRCA1 and BRCA2 mutations. Journal of Clinical Oncology.

[86] Sakai W, Swisher EM, Karlan BY, Agarwal MK, Higgins J, Friedman C, Villegas E, Jacquemont C, Farrugia DJ, Couch FJ (2008). Secondary mutations as a mechanism of cisplatin resistance in BRCA2-mutated cancers. Nature.

[87] Cheung LW, Hennessy BT, Li J, Yu S, Myers AP, Djordjevic B, Lu Y, Stemke-Hale K, Dyer MD, Zhang F (2011). High frequency of PIK3R1 and PIK3R2 mutations in endometrial cancer elucidates a novel mechanism for regulation of PTEN protein stability. Cancer discovery.

[88] Urick ME, Rudd ML, Godwin AK, Sgroi D, Merino M, Bell DW (2011). PIK3R1 (p85α) is somatically mutated at high frequency in primary endometrial cancer. Cancer research.

[89] Rudd ML, Price JC, Fogoros S, Godwin AK, Sgroi DC, Merino MJ, Bell DW (2011). A unique spectrum of somatic PIK3CA (p110α) mutations within primary endometrial carcinomas. Clinical Cancer Research.

[90] Campbell IG, Russell SE, Choong DY, Montgomery KG, Ciavarella ML, Hooi CS, Cristiano BE, Pearson RB, Phillips WA (2004). Mutation of the PIK3CA gene in ovarian and breast cancer. Cancer research.

[91] Oda K, Stokoe D, Taketani Y, McCormick F (2005). High frequency of coexistent mutations of PIK3CA and PTEN genes in endometrial carcinoma. Cancer research.

[92] Cerami E, Gao J, Dogrusoz U, Gross BE, Sumer SO, Aksoy BA, Jacobsen A, Byrne CJ, Heuer ML, Larsson E, Antipin Y, Reva B, Goldberg AP (2012). The cBio Cancer Genomics Portal: An Open Platform for Exploring Multidimensional Cancer Genomics Data. Cancer Discovery.

[93] Fabi F, Asselin E (2014). Expression, activation, and role of AKT isoforms in the uterus. Reproduction.

[94] Lee S, Choi E-J, Jin C, Kim D-H (2005). Activation of PI3K/Akt pathway by PTEN reduction and PIK3CA mRNA amplification contributes to cisplatin resistance in an ovarian cancer cell line. Gynecologic oncology.

[95] Mitsuuchi Y, Johnson SW, Selvakumaran M, Williams SJ, Hamilton TC, Testa JR (2000). The phosphatidylinositol 3-kinase/AKT signal transduction pathway plays a critical role in the expression of p21WAF1/CIP1/SDI1 induced by cisplatin and paclitaxel. Cancer Research.

[96] Gagnon V, Mathieu I, Sexton E, Leblanc K, Asselin E (2004). AKT involvement in cisplatin chemoresistance of human uterine cancer cells. Gynecol Oncol.

[97] Gagnon V, Van Themsche C, Turner S, Leblanc V, Asselin E (2008). Akt and XIAP regulate the sensitivity of human uterine cancer cells to cisplatin, doxorubicin and taxol. Apoptosis.

[98] Girouard J, Lafleur M-J, Parent S, Leblanc V, Asselin E (2013). Involvement of Akt isoforms in chemoresistance of endometrial carcinoma cells. Gynecologic Oncology.

[99] Fraser M, Leung BM, Yan X, Dan HC, Cheng JQ, Tsang BK (2003). p53 Is a Determinant of X-Linked Inhibitor of Apoptosis Protein/Akt-Mediated Chemoresistance in Human Ovarian Cancer Cells. Cancer Research.

[100] Asselin E, Mills GB, Tsang BK (2001). XIAP Regulates Akt Activity and Caspase-3-dependent Cleavage during Cisplatin-induced Apoptosis in Human Ovarian Epithelial Cancer Cells. Cancer Research.

[101] Yang X, Fraser M, Moll UM, Basak A, Tsang BK (2006). Akt-mediated cisplatin resistance in ovarian cancer: modulation of p53 action on caspase-dependent mitochondrial death pathway. Cancer research.

[102] Abedini M, Muller E, Bergeron R, Gray D, Tsang B (2010). Akt promotes chemoresistance in human ovarian cancer cells by modulating cisplatin-induced, p53-dependent ubiquitination of FLICE-like inhibitory protein. Oncogene.

[103] Kim SH, Juhnn YS, Song YS (2007). Akt involvement in paclitaxel chemoresistance of human ovarian cancer cells. Annals of the New York Academy of Sciences.

[104] Peng D-J, Wang J, Zhou J-Y, Wu GS (2010). Role of the Akt/mTOR survival pathway in cisplatin resistance in ovarian cancer cells. Biochemical and biophysical research communications.

[105] Yuan Z-q, Feldman RI, Sussman GE, Coppola D, Nicosia SV, Cheng JQ (2003). AKT2 Inhibition of Cisplatin-induced JNK/p38 and Bax Activation by Phosphorylation of ASK1 IMPLICATION OF AKT2 IN CHEMORESISTANCE. Journal of Biological Chemistry.

[106] Yan X, Fraser M, Qiu Q, Tsang BK (2006). Over-expression of PTEN sensitizes human ovarian cancer cells to cisplatin-induced apoptosis in a p53-dependent manner. Gynecologic oncology.

[107] Tashiro H, Blazes MS, Wu R, Cho KR, Bose S, Wang SI, Li J, Parsons R, Ellenson LH (1997). Mutations in PTEN are frequent in endometrial carcinoma but rare in other common gynecological malignancies. Cancer research.

[108] Wu H, Cao Y, Weng D, Xing H, Song X, Zhou J, Xu G, Lu Y, Wang S, Ma D (2008). Effect of tumor suppressor gene PTEN on the resistance to cisplatin in human ovarian cancer cell lines and related mechanisms. Cancer letters.

[109] Singh M, Chaudhry P, Fabi F, Asselin E (2013). Cisplatin-induced caspase activation mediates PTEN cleavage in ovarian cancer cells: a potential mechanism of chemoresistance. BMC cancer.

[110] Ying H, Qu D, Liu C, Ying T, Lv J, Jin S, Xu H (2015). Chemoresistance is associated with Beclin-1 and PTEN expression in epithelial ovarian cancers. Oncology letters.

[111] Van Themsche C, Leblanc V, Parent S, Asselin E (2009). X-linked inhibitor of apoptosis protein (XIAP) regulates PTEN ubiquitination, content, and compartmentalization. J Biol Chem.

[112] Asselin E, Wang Y, Tsang BK (2001). X-Linked Inhibitor of Apoptosis Protein Activates the Phosphatidylinositol 3-Kinase/Akt Pathway in Rat Granulosa Cells during Follicular Development 1. Endocrinology.

[113] Cheng JQ, Jiang X, Fraser M, Li M, Dan HC, Sun M, Tsang BK (2002). Role of X-linked inhibitor of apoptosis protein in chemoresistance in ovarian cancer: possible involvement of the phosphoinositide-3 kinase/Akt pathway. Drug resistance updates.

[114] Dan HC, Sun M, Kaneko S, Feldman RI, Nicosia SV, Wang H-G, Tsang BK, Cheng JQ (2004). Akt Phosphorylation and Stabilization of X-linked Inhibitor of Apoptosis Protein (XIAP). Journal of Biological Chemistry.

[115] Mansouri A, Zhang Q, Ridgway LD, Tian L, Claret F-X (2003). Cisplatin resistance in an ovarian carcinoma is associated with a defect in programmed cell death control through XIAP regulation. Oncology research.

[116] Ma J-j, Chen B-l (2009). Xin X-y. XIAP gene downregulation by small interfering RNA inhibits proliferation, induces apoptosis, and reverses the cisplatin resistance of ovarian carcinoma. European Journal of Obstetrics & Gynecology and Reproductive Biology.

[117] Sasaki H, Sheng Y, Kotsuji F, Tsang BK (2000). Down-regulation of X-linked inhibitor of apoptosis protein induces apoptosis in chemoresistant human ovarian cancer cells. Cancer Research.

[118] Li J, Sasaki H, Sheng Y, Schneiderman D, Xiao C, Kotsuji F, Tsang BK (2000). Apoptosis and chemoresistance in human ovarian cancer: is Xiap a determinant?. Neurosignals.

[119] Astanehe A, Arenillas D, Wasserman WW, Leung PC, Dunn SE, Davies BR, Mills GB, Auersperg N (2008). Mechanisms underlying p53 regulation of PIK3CA transcription in ovarian surface epithelium and in ovarian cancer. Journal of cell science.

[120] Fraser M, Bai T, Tsang BK (2008). Akt promotes cisplatin resistance in human ovarian cancer cells through inhibition of p53 phosphorylation and nuclear function. International Journal of Cancer.

[121] Roberts P, Der C (2007). Targeting the Raf-MEK-ERK mitogen-activated protein kinase cascade for the treatment of cancer. Oncogene.

[122] Pearson G, Robinson F, Beers Gibson T, Xu B-e, Karandikar M, Berman K, Cobb MH (2001). Mitogen-activated protein (MAP) kinase pathways: regulation and physiological functions 1. Endocrine reviews.

[123] Lu Z, Xu S (2006). ERK1/2 MAP kinases in cell survival and apoptosis. IUBMB Life.

[124] Cagnol S, Chambard J-C (2010). ERK and cell death: Mechanisms of ERK-induced cell death – apoptosis, autophagy and senescence. FEBS Journal.

[125] Mansouri A, Ridgway LD, Korapati AL, Zhang Q, Tian L, Wang Y, Siddik ZH, Mills GB, Claret FX (2003). Sustained activation of JNK/p38 MAPK pathways in response to cisplatin leads to Fas ligand induction and cell death in ovarian carcinoma cells. Journal of Biological Chemistry.

[126] Villedieu M, Deslandes E, Duval M, Héron J-F, Gauduchon P, Poulain L (2006). Acquisition of chemoresistance following discontinuous exposures to cisplatin is associated in ovarian carcinoma cells with progressive alteration of FAK, ERK and p38 activation in response to treatment. Gynecologic oncology.

[127] Chan DW, Liu VW, Tsao GS, Yao K-M, Furukawa T, Chan KK, Ngan HY (2008). Loss of MKP3 mediated by oxidative stress enhances tumorigenicity and chemoresistance of ovarian cancer cells. Carcinogenesis.

[128] Mendoza MC, Er EE, Blenis J (2011). The Ras-ERK and PI3K-mTOR pathways: cross-talk and compensation. Trends in Biochemical Sciences.

[129] Villella J, Cohen S, Smith D, Hibshoosh H, Hershman D (2006). HER‐2/neu overexpression in uterine papillary serous cancers and its possible therapeutic implications. International Journal of Gynecological Cancer.

[130] Slomovitz BM, Broaddus RR, Burke TW, Sneige N, Soliman PT, Wu W, Sun CC, Munsell MF, Gershenson DM, Lu KH (2004). Her-2/neu overexpression and amplification in uterine papillary serous carcinoma. Journal of Clinical Oncology.

[131] Nicholson RI, Gee JMW, Harper ME (2001). EGFR and cancer prognosis. European journal of cancer.

[132] Konecny G, Santos L, Winterhoff B, Hatmal M, Keeney G, Mariani A, Jones M, Neuper C, Thomas B, Muderspach L (2009). HER2 gene amplification and EGFR expression in a large cohort of surgically staged patients with nonendometrioid (type II) endometrial cancer. British journal of cancer.

[133] Sheng Q, Liu J (2011). The therapeutic potential of targeting the EGFR family in epithelial ovarian cancer. British journal of cancer.

[134] Stadlmann S, Gueth U, Reiser U, Diener P-A, Zeimet AG, Wight E, Mirlacher M, Sauter G, Mihatsch MJ, Singer G (2006). Epithelial growth factor receptor status in primary and recurrent ovarian cancer. Modern Pathology.

[135] Siwak DR, Carey M, Hennessy BT, Nguyen CT, McGahren Murray MJ, Nolden L, Mills GB (2009). Targeting the epidermal growth factor receptor in epithelial ovarian cancer: current knowledge and future challenges. Journal of oncology.

[136] Reyes HD, Thiel KW, Carlson MJ, Meng X, Yang S, Stephan J-M, Leslie KK (2014). Comprehensive profiling of EGFR/HER receptors for personalized treatment of gynecologic cancers. Molecular diagnosis & therapy.

[137] Marth C, Widschwendter M, Kaern J (1997). Cisplatin resistance is associated with reduced interferon-gamma-sensitivity and increased HER-2 expression in cultured ovarian carcinoma cells. British journal of cancer.

[138] Pegram MD, Finn RS, Arzoo K, Beryt M, Pietras RJ, Slamon DJ (1997). The effect of HER-2/neu overexpression on chemotherapeutic drug sensitivity in human breast and ovarian cancer cells. Oncogene.

[139] Mori N, Kyo S, Nakamura M, Hashimoto M, Maida Y, Mizumoto Y, Takakura M, Ohno S, Kiyono T, Inoue M (2010). Expression of HER-2 affects patient survival and paclitaxel sensitivity in endometrial cancer. British journal of cancer.

[140] Hengstler JG, Lange J, Kett A, Dornhöfer N, Meinert R, Arand M, Knapstein PG, Becker R, Oesch F, Tanner B (1999). Contribution of c-erbB-2 and topoisomerase IIα to chemoresistance in ovarian cancer. Cancer research.

[141] Qiu L, Jiang Q, Di W, Scheffler E, Derby S, Yang J, Kouttab N, Wanebo H, Yan B, Wan Y (2005). Targeted inhibition of transient activation of the EGFR-mediated cell survival pathway enhances paclitaxel-induced ovarian cancer cell death. International journal of oncology.

[142] Sasaki N, Kudoh K, Kita T, Tsuda H, Furuya K, Kikuchi Y (2007). Effect of HER‐2/neu overexpression on chemoresistance and prognosis in ovarian carcinoma. Journal of Obstetrics and Gynaecology Research.

[143] Pearce ST, Jordan VC (2004). The biological role of estrogen receptors [alpha] and [beta] in cancer. Critical Reviews in Oncology/Hematology.

[144] Wray S, Noble K (2008). Sex hormones and excitation-contraction coupling in the uterus: the effects of oestrous and hormones. J Neuroendocrinol.

[145] Dauvois S, White R, Parker MG (1993). The antiestrogen ICI 182780 disrupts estrogen receptor nucleocytoplasmic shuttling. J Cell Sci.

[146] Bjornstrom L, Sjoberg M (2005). Mechanisms of estrogen receptor signaling: convergence of genomic and nongenomic actions on target genes. Molecular endocrinology.

[147] Prossnitz ER, Arterburn JB, Sklar LA (2007). GPR30: AG protein-coupled receptor for estrogen. Molecular and cellular endocrinology.

[148] Won YS, Lee SJ, Yeo SG, Park DC (2012). Effects of female sex hormones on clusterin expression and paclitaxel resistance in endometrial cancer cell lines. Int J Med Sci.

[149] Luvsandagva B, Nakamura K, Kitahara Y, Aoki H, Murata T, Ikeda S, Minegishi T (2012). GRP78 induced by estrogen plays a role in the chemosensitivity of endometrial cancer. Gynecologic Oncology.

[150] Mabuchi S, Ohmichi M, Kimura A, Nishio Y, Arimoto-Ishida E, Yada-Hashimoto N, Tasaka K, Murata Y (2004). Estrogen inhibits paclitaxel-induced apoptosis via the phosphorylation of apoptosis signal-regulating kinase 1 in human ovarian cancer cell lines. Endocrinology.

[151] Sui M, Zhang H, Fan W (2011). The Role of Estrogen and Estrogen Receptors in Chemoresistance. Curr Med Chem.

[152] Bieging KT, Mello SS, Attardi LD (2014). Unravelling mechanisms of p53-mediated tumour suppression. Nature Reviews Cancer.

[153] Lane DP (1992). Cancer. p53, guardian of the genome. Nature.

[154] Berchuck A, Kohler MF, Marks JR, Wiseman R, Boyd J, Bast RC (1994). The p53 tumor suppressor gene frequently is altered in gynecologic cancers. American journal of obstetrics and gynecology.

[155] Garg K, Leitao MM, Wynveen CA, Sica GL, Shia J, Shi W, Soslow RA (2010). p53 overexpression in morphologically ambiguous endometrial carcinomas correlates with adverse clinical outcomes. Modern Pathology.

[156] Nijman H, Lambeck A, Van Der Burg S, Van Der Zee A, Daemen T (2005). Immunologic aspect of ovarian cancer and p53 as tumor antigen. Journal of translational medicine.

[157] Kurrey NK, Jalgaonkar SP, Joglekar AV, Ghanate AD, Chaskar PD, Doiphode RY, Bapat SA (2009). Snail and Slug Mediate Radioresistance and Chemoresistance by Antagonizing p53‐Mediated Apoptosis and Acquiring a Stem‐Like Phenotype in Ovarian Cancer Cells. Stem cells.

[158] Reles A, Wen WH, Schmider A, Gee C, Runnebaum IB, Kilian U, Jones LA, El-Naggar A, Minguillon C, Schönborn I (2001). Correlation of p53 mutations with resistance to platinum-based chemotherapy and shortened survival in ovarian cancer. Clinical Cancer Research.

[159] Buttitta F, Marchetti A, Gadducci A, Pellegrini S, Morganti M, Carnicelli V, Cosio S, Gagetti O, Genazzani A, Bevilacqua G (1997). p53 alterations are predictive of chemoresistance and aggressiveness in ovarian carcinomas: a molecular and immunohistochemical study. British journal of cancer.

[160] Leung E, Fraser M, Fiscus R, Tsang B (2008). Cisplatin alters nitric oxide synthase levels in human ovarian cancer cells: involvement in p53 regulation and cisplatin resistance. British journal of cancer.

[161] Abedini MR, Muller EJ, Brun J, Bergeron R, Gray DA, Tsang BK (2008). Cisplatin induces p53-dependent FLICE-like inhibitory protein ubiquitination in ovarian cancer cells. Cancer research.

[162] Sato S, Kigawa J, Minagawa Y, Okada M, Shimada M, Takahashi M, Kamazawa S, Terakawa N (1999). Chemosensitivity and p53‐dependent apoptosis in epithelial ovarian carcinoma. Cancer.

[163] Jones NA, Turner J, McIlwrath AJ, Brown R, Dive C (1998). Cisplatin-and paclitaxel-induced apoptosis of ovarian carcinoma cells and the relationship between bax and bak up-regulation and the functional status of p53. Molecular pharmacology.

[164] Mir R, Tortosa A, Martinez‐Soler F, Vidal A, Condom E, Pérez‐Perarnau A, Ruiz‐Larroya T, Gil J, Giménez‐Bonafé P (2013). Mdm2 antagonists induce apoptosis and synergize with cisplatin overcoming chemoresistance in TP53 wild‐type ovarian cancer cells. International Journal of Cancer.

[165] Lavarino C, Delia D, Di Palma S, Zunino F, Pilotti S (1997). p53 in drug resistance in ovarian cancer. The Lancet.

[166] Vandenput I, Capoen A, Coenegrachts L, Verbist G, Moerman P, Vergote I, Amant F (2011). Expression of ERCC1, p53, and class III β-tubulin do not reveal chemoresistance in endometrial cancer: results from an immunohistochemical study. International Journal of Gynecological Cancer.

[167] Sultana H, Kigawa J, Kanamori Y, Itamochi H, Oishi T, Sato S, Kamazawa S, Ohwada M, Suzuki M, Terakawa N (2003). Chemosensitivity and p53–Bax pathway-mediated apoptosis in patients with uterine cervical cancer. Annals of oncology.

[168] Rouette A, Parent S, Girouard J, Leblanc V, Asselin E (2012). Cisplatin increases B-cell-lymphoma-2 expression via activation of protein kinase C and Akt2 in endometrial cancer cells. International Journal of Cancer.

[169] Perego P, Righetti S, Supino R, Delia D, Caserini C, Carenini N, Bedogne B, Broome E, Krajewski S, Reed J (1997). Role of apoptosis and apoptosis-related proteins in the cisplatin-resistant phenotype of human tumor cell lines. Apoptosis.

[170] El-Guendy N, Zhao Y, Gurumurthy S, Burikhanov R, Rangnekar VM (2003). Identification of a Unique Core Domain of Par-4 Sufficient for Selective Apoptosis Induction in Cancer Cells. Molecular and Cellular Biology.

[171] Shrestha-Bhattarai T, Rangnekar VM (2010). Cancer-selective apoptotic effects of extracellular and intracellular Par-4. Oncogene.

[172] Tan J, You Y, Xu T, Yu P, Wu D, Deng H, Zhang Y, Bie P (2014). Par-4 downregulation confers cisplatin resistance in pancreatic cancer cells via PI3K/Akt pathway-dependent EMT. Toxicology Letters.

[173] Boehrer S, Chow KU, Beske F, Kukoc-Zivojnov N, Puccetti E, Ruthardt M, Baum C, Rangnekar VM, Hoelzer D, In Mitrou PS (2002). lymphatic cells par-4 sensitizes to apoptosis by down-regulating bcl-2 and promoting disruption of mitochondrial membrane potential and caspase activation. Cancer research.

[174] Jagtap JC, Dawood P, Shah RD, Chandrika G, Natesh K, Shiras A, Hegde AS, Ranade D, Shastry P (2014). Expression and Regulation of Prostate Apoptosis Response-4 (Par-4) in Human Glioma Stem Cells in Drug-Induced Apoptosis. PLoS ONE.

[175] Pereira MC, De Bessa-Garcia SA, Burikhanov R, Pavanelli AC, Antunes L, Rangnekar VM, Nagai MA (2013). Prostate apoptosis response-4 is involved in the apoptosis response to docetaxel in MCF-7 breast cancer cells. International Journal of Oncology.

[176] Jagtap JC, Parveen D, Shah RD, Desai A, Bhosale D, Chugh A, Ranade D, Karnik S, Khedkar B, Mathur A (2015). Secretory prostate apoptosis response (Par)‐4 sensitizes multicellular spheroids (MCS) of glioblastoma multiforme cells to tamoxifen‐induced cell death. FEBS open bio.

[177] Casolari DA, Pereira MC, de Bessa Garcia SA, Nagai MA (2011). Insulin-like growth factor-1 and 17beta-estradiol down-regulate prostate apoptosis response-4 expression in MCF-7 breast cancer cells. Int J Mol Med.

[178] Brasseur K, Fabi F, Adam P, Parent S, Lessard L, Asselin E (2016). Post-translational regulation of the cleaved fragment of Par-4 in ovarian and endometrial cancer cells. Oncotarget.

[179] Meynier S, Kramer M, Ribaux P, Tille JC, Delie F, Petignat P, Cohen M (2015). Role of PAR-4 in ovarian cancer. Oncotarget.

[180] Leonard GD, Fojo T, Bates SE (2003). The role of ABC transporters in clinical practice. The oncologist.

[181] Seiden MV, Swenerton KD, Matulonis U, Campos S, Rose P, Batist G, Ette E, Garg V, Fuller A, Harding MW (2002). A phase II study of the MDR inhibitor biricodar (INCEL, VX-710) and paclitaxel in women with advanced ovarian cancer refractory to paclitaxel therapy. Gynecologic oncology.

[182] Fracasso PM, Brady MF, Moore DH, Walker JL, Rose PG, Letvak L, Grogan TM, McGuire WP (2001). Phase II study of paclitaxel and valspodar (PSC 833) in refractory ovarian carcinoma: a gynecologic oncology group study. Journal of clinical oncology.

[183] Kelly RJ, Draper D, Chen CC, Robey RW, Figg WD, Piekarz RL, Chen X, Gardner ER, Balis FM, Venkatesan AM (2011). A pharmacodynamic study of docetaxel in combination with the P-glycoprotein antagonist tariquidar (XR9576) in patients with lung, ovarian, and cervical cancer. Clinical Cancer Research.

[184] Mistry P, Stewart AJ, Dangerfield W, Okiji S, Liddle C, Bootle D, Plumb JA, Templeton D, Charlton P (2001). In vitro and in vivo reversal of P-glycoprotein-mediated multidrug resistance by a novel potent modulator, XR9576. Cancer research.

[185] Di Nicolantonio F, Knight LA, Glaysher S, Whitehouse PA, Mercer SJ, Sharma S, Mills L, Prin A, Johnson P, Charlton PA (2004). Ex vivo reversal of chemoresistance by tariquidar (XR9576). Anti-cancer drugs.

[186] Tutt A, Lord C, McCabe N, Farmer H, Turner N, Martin N, Jackson S, Smith G, Ashworth A (2005). Exploiting the DNA repair defect in BRCA mutant cells in the design of new therapeutic strategies for cancer. Cold Spring Harbor Symposia on Quantitative Biology.

[187] Audeh MW, Carmichael J, Penson RT, Friedlander M, Powell B, Bell-McGuinn KM, Scott C, Weitzel JN, Oaknin A, Loman N (2010). Oral poly (ADP-ribose) polymerase inhibitor olaparib in patients with BRCA1 or BRCA2 mutations and recurrent ovarian cancer: a proof-of-concept trial. The Lancet.

[188] Ledermann J, Harter P, Gourley C, Friedlander M, Vergote I, Rustin G, Scott C, Meier W, Shapira-Frommer R, Safra T (2012). Olaparib maintenance therapy in platinum-sensitive relapsed ovarian cancer. New England Journal of Medicine.

[189] Gelmon KA, Tischkowitz M, Mackay H, Swenerton K, Robidoux A, Tonkin K, Hirte H, Huntsman D, Clemons M, Gilks B (2011). Olaparib in patients with recurrent high-grade serous or poorly differentiated ovarian carcinoma or triple-negative breast cancer: a phase 2, multicentre, open-label, non-randomised study. The lancet oncology.

[190] Kaufman B, Shapira-Frommer R, Schmutzler RK, Audeh MW, Friedlander M, Balmaña J, Mitchell G, Fried G, Stemmer SM, Hubert A (2015). Olaparib monotherapy in patients with advanced cancer and a germline BRCA1/2 mutation. Journal of Clinical Oncology.

[191] Lee J-M, Hays JL, Annunziata CM, Noonan AM, Minasian L, Zujewski JA, Yu M, Gordon N, Ji J, Sissung TM (2014). Phase I/Ib study of olaparib and carboplatin in BRCA1 or BRCA2 mutation-associated breast or ovarian cancer with biomarker analyses. Journal of the National Cancer Institute.

[192] Brunet A, Bonni A, Zigmond MJ, Lin MZ, Juo P, Hu LS, Anderson MJ, Arden KC, Blenis J, Greenberg ME (1999). Akt promotes cell survival by phosphorylating and inhibiting a Forkhead transcription factor. cell.

[193] Oza AM, Cibula D, Benzaquen AO, Poole C, Mathijssen RH, Sonke GS, Colombo N, Špaček J, Vuylsteke P, Hirte H (2015). Olaparib combined with chemotherapy for recurrent platinum-sensitive ovarian cancer: a randomised phase 2 trial. The Lancet Oncology.

[194] Liu JF, Barry WT, Birrer M, Lee J-M, Buckanovich RJ, Fleming GF, Rimel B, Buss MK, Nattam S, Hurteau J (2014). Combination cediranib and olaparib versus olaparib alone for women with recurrent platinum-sensitive ovarian cancer: a randomised phase 2 study. The Lancet Oncology.

[195] Coleman RL, Sill MW, Bell-McGuinn K, Aghajanian C, Gray HJ, Tewari KS, Rubin SC, Rutherford TJ, Chan JK, Chen A (2015). A phase II evaluation of the potent, highly selective PARP inhibitor veliparib in the treatment of persistent or recurrent epithelial ovarian, fallopian tube, or primary peritoneal cancer in patients who carry a germline BRCA1 or BRCA2 mutation—An NRG Oncology/Gynecologic Oncology Group study. Gynecologic oncology.

[196] Kummar S, Oza AM, Fleming GF, Sullivan DM, Gandara DR, Naughton MJ, Villalona-Calero MA, Morgan RJ, Szabo PM, Youn A (2015). Randomized trial of oral cyclophosphamide and veliparib in high-grade serous ovarian, primary peritoneal, or fallopian tube cancers, or BRCA-mutant ovarian cancer. Clinical Cancer Research.

[197] Sandhu SK, Schelman WR, Wilding G, Moreno V, Baird RD, Miranda S, Hylands L, Riisnaes R, Forster M, Omlin A (2013). The poly (ADP-ribose) polymerase inhibitor niraparib (MK4827) in BRCA mutation carriers and patients with sporadic cancer: a phase 1 dose-escalation trial. The lancet oncology.

[198] Penson R, Whalen C, Lasonde B, Krasner C, Konstantinopoulos P, Stallings T, Bradley C, Birrer M, Matulonis U (2011). A phase II trial of iniparib (BSI-201) in combination with gemcitabine/carboplatin (GC) in patients with platinum-sensitive recurrent ovarian cancer. ASCO Annual Meeting Proceedings.

[199] Aghajanian C, Sill MW, Secord AA, Powell MA, Steinhoff M (2012). Iniparib plus paclitaxel and carboplatin as initial treatment of advanced or recurrent uterine carcinosarcoma: a Gynecologic Oncology Group Study. Gynecologic oncology.

[200] Ihnen M, zu Eulenburg C, Kolarova T, Qi JW, Manivong K, Chalukya M, Dering J, Anderson L, Ginther C, Meuter A (2013). Therapeutic potential of the poly (ADP-ribose) polymerase inhibitor rucaparib for the treatment of sporadic human ovarian cancer. Molecular cancer therapeutics.

[201] Kristeleit RS, Shapiro G, LoRusso P, Infante JR, Flynn M, Patel MR, Tolaney SM, Hilton JF, Calvert AH, Giordano H (2013). A phase I dose-escalation and PK study of continuous oral rucaparib in patients with advanced solid tumors. ASCO Annual Meeting Proceedings.

[202] Drew Y, Ledermann J, Hall G, Rea D, Glasspool R, Highley M, Jayson G, Sludden J, Murray J, Jamieson D (2016). Phase 2 multicentre trial investigating intermittent and continuous dosing schedules of the poly (ADP-ribose) polymerase inhibitor rucaparib in germline BRCA mutation carriers with advanced ovarian and breast cancer. British Journal of Cancer.

[203] Yin Y, Shen W (2008). PTEN: a new guardian of the genome. Oncogene.

[204] Miyasaka A, Oda K, Ikeda Y, Wada-Hiraike O, Kashiyama T, Enomoto A, Hosoya N, Koso T, Fukuda T, Inaba K (2014). Anti-tumor activity of olaparib, a poly (ADP-ribose) polymerase (PARP) inhibitor, in cultured endometrial carcinoma cells. BMC cancer.

[205] Mendes‐Pereira AM, Martin SA, Brough R, McCarthy A, Taylor JR, Kim JS, Waldman T, Lord CJ, Ashworth A (2009). Synthetic lethal targeting of PTEN mutant cells with PARP inhibitors. EMBO molecular medicine.

[206] Forster MD, Dedes KJ, Sandhu S, Frentzas S, Kristeleit R, Ashworth A, Poole CJ, Weigelt B, Kaye SB, Molife LR (2011). Treatment with olaparib in a patient with PTEN-deficient endometrioid endometrial cancer. Nature Reviews Clinical Oncology.

[207] Gentile F, Tuszynski JA, Barakat KH (2016). New design of nucleotide excision repair (NER) inhibitors for combination cancer therapy. Journal of Molecular Graphics and Modelling.

[208] Martin SA, Hewish M, Sims D, Lord CJ, Ashworth A (2011). Parallel high-throughput RNA interference screens identify PINK1 as a potential therapeutic target for the treatment of DNA mismatch repair–deficient cancers. Cancer research.

[209] Martin SA, McCabe N, Mullarkey M, Cummins R, Burgess DJ, Nakabeppu Y, Oka S, Kay E, Lord CJ, Ashworth A (2010). DNA polymerases as potential therapeutic targets for cancers deficient in the DNA mismatch repair proteins MSH2 or MLH1. Cancer cell.

[210] Martin SA, McCarthy A, Barber LJ, Burgess DJ, Parry S, Lord CJ, Ashworth A (2009). Methotrexate induces oxidative DNA damage and is selectively lethal to tumour cells with defects in the DNA mismatch repair gene MSH2. EMBO molecular medicine.

[211] Xiao X, Melton DW, Gourley C (2014). Mismatch repair deficiency in ovarian cancer—molecular characteristics and clinical implications. Gynecologic oncology.

[212] Plumb JA, Strathdee G, Sludden J, Kaye SB, Brown R (2000). Reversal of drug resistance in human tumor xenografts by 2′-deoxy-5-azacytidine-induced demethylation of the hMLH1 gene promoter. Cancer research.

[213] Fang F, Balch C, Schilder J, Breen T, Zhang S, Shen C, Li L, Kulesavage C, Snyder AJ, Nephew KP (2010). A phase 1 and pharmacodynamic study of decitabine in combination with carboplatin in patients with recurrent, platinum‐resistant, epithelial ovarian cancer. Cancer.

[214] Matei D, Fang F, Shen C, Schilder J, Arnold A, Zeng Y, Berry WA, Huang T, Nephew KP (2012). Epigenetic resensitization to platinum in ovarian cancer. Cancer research.

[215] Ohta T, Ohmichi M, Hayasaka T, Mabuchi S, Saitoh M, Kawagoe J, Takahashi K, Igarashi H, Du B, Doshida M (2006). Inhibition of phosphatidylinositol 3-kinase increases efficacy of cisplatin in in vivo ovarian cancer models. Endocrinology.

[216] Juvekar A, Burga LN, Hu H, Lunsford EP, Ibrahim YH, Balmañà J, Rajendran A, Papa A, Spencer K, Lyssiotis CA (2012). Combining a PI3K inhibitor with a PARP inhibitor provides an effective therapy for BRCA1-related breast cancer. Cancer discovery.

[217] Matulonis U, Wulf GM, Birrer MJ, Westin SN, Quy P, Bell-McGuinn KM, Lasonde B, Whalen C, Aghajanian C, Solit DB (2014). Phase I study of oral BKM120 and oral olaparib for high-grade serous ovarian cancer (HGSC) or triple-negative breast cancer (TNBC). ASCO Annual Meeting Proceedings.

[218] Von Hoff D, LoRusso P, Demetri G, Weiss G, Shapiro G, Ramanathan R, Ware J, Raja R, Jin J, Levy G (2011). A phase I dose-escalation study to evaluate GDC-0941, a pan-PI3K inhibitor, administered QD or BID in patients with advanced or metastatic solid tumors. ASCO annual meeting proceedings.

[219] Moreno Garcia V, Baird R, Shah K, Basu B, Tunariu N, Blanco M, Cassier P, Pedersen J, Puglisi M, Sarker D (2011). A phase I study evaluating GDC-0941, an oral phosphoinositide-3 kinase (PI3K) inhibitor, in patients with advanced solid tumors or multiple myeloma. ASCO annual meeting proceedings.

[220] Matulonis U, Vergote I, Backes F, Martin LP, McMeekin S, Birrer M, Campana F, Xu Y, Egile C, Ghamande S (2015). Phase II study of the PI3K inhibitor pilaralisib (SAR245408; XL147) in patients with advanced or recurrent endometrial carcinoma. Gynecologic oncology.

[221] Gonzalez-Angulo AM, Juric D, Argiles G, Schellens JH, Burris HA, Berlin J, Middleton MR, Schuler MH, Geel RV, Helgason T (2013). Safety, pharmacokinetics, and preliminary activity of the {alpha}-specific PI3K inhibitor BYL719: Results from the first-in-human study. ASCO Annual Meeting Proceedings.

[222] Kigawa J (2013). New Strategy for Overcoming Resistance to Chemotherapy of Ovarian Cancer. Yonago Acta medica.

[223] Sinha D, Bannergee S, Schwartz JH, Lieberthal W, Levine JS (2004). Inhibition of ligand-independent ERK1/2 activity in kidney proximal tubular cells deprived of soluble survival factors up-regulates Akt and prevents apoptosis. Journal of Biological Chemistry.

[224] Bedard PL, Tabernero J, Janku F, Wainberg ZA, Paz-Ares L, Vansteenkiste J, Van Cutsem E, Pérez-García J, Stathis A, Britten CD (2015). A phase Ib dose-escalation study of the oral pan-PI3K inhibitor buparlisib (BKM120) in combination with the oral MEK1/2 inhibitor trametinib (GSK1120212) in patients with selected advanced solid tumors. Clinical Cancer Research.

[225] Juric D, Soria J-C, Sharma S, Banerji U, Azaro A, Desai J, Ringeisen FP, Kaag A, Radhakrishnan R, Hourcade-Potelleret F (2014). A phase 1b dose-escalation study of BYL719 plus binimetinib (MEK162) in patients with selected advanced solid tumors. ASCO Annual Meeting Proceedings.

[226] Meyer LA, Slomovitz BM, Djordjevic B, Westin SN, Iglesias DA, Munsell MF, Jiang Y, Schmandt R, Broaddus RR, Coleman RL (2014). The search continues: looking for predictive biomarkers for response to mammalian target of rapamycin inhibition in endometrial cancer. International journal of gynecological cancer.

[227] Santiskulvong C, Konecny GE, Fekete M, Chen K-YM, Karam A, Mulholland D, Eng C, Wu H, Song M, Dorigo O (2011). Dual targeting of phosphoinositide 3-kinase and mammalian target of rapamycin using NVP-BEZ235 as a novel therapeutic approach in human ovarian carcinoma. Clinical Cancer Research.

[228] Shoji K, Oda K, Kashiyama T, Ikeda Y, Nakagawa S, Sone K, Miyamoto Y, Hiraike H, Tanikawa M, Miyasaka A (2012). Genotype-dependent efficacy of a dual PI3K/mTOR inhibitor, NVP-BEZ235, and an mTOR inhibitor, RAD001, in endometrial carcinomas. PloS one.

[229] Lin Y-H, Chen BY-H, Lai W-T, Wu S-F, Guh J-H, Cheng A-L, Hsu L-C (2015). The Akt inhibitor MK-2206 enhances the cytotoxicity of paclitaxel (Taxol) and cisplatin in ovarian cancer cells. Naunyn-Schmiedeberg’s archives of pharmacology.

[230] Pant A, Lee II, Lu Z, Rueda BR, Schink J, Kim JJ (2012). Inhibition of AKT with the orally active allosteric AKT inhibitor, MK-2206, sensitizes endometrial cancer cells to progestin. PLoS One.

[231] Engel JB, Honig A, Schönhals T, Weidler C, Häusler S, Krockenberger M, Grunewald TG, Dombrowski Y, Rieger L, Dietl J (2008). Perifosine inhibits growth of human experimental endometrial cancers by blockade of AKT phosphorylation. European Journal of Obstetrics & Gynecology and Reproductive Biology.

[232] Sun H, Yu T, Li J (2011). Co-administration of perifosine with paclitaxel synergistically induces apoptosis in ovarian cancer cells: more than just AKT inhibition. Cancer letters.

[233] Engel JB, Schönhals T, Häusler S, Krockenberger M, Schmidt M, Horn E, Köster F, Dietl J, Wischhusen J, Honig A (2011). Induction of programmed cell death by inhibition of AKT with the alkylphosphocholine perifosine in in vitro models of platinum sensitive and resistant ovarian cancers. Archives of gynecology and obstetrics.

[234] Hahne J, Honig A, Meyer S, Gambaryan S, Walter U, Wischhusen J, Häussler S, Segerer S, Fujita N, Dietl J (2012). Downregulation of AKT reverses platinum resistance of human ovarian cancers in vitro. Oncology reports.

[235] Davies BR, Greenwood H, Dudley P, Crafter C, Yu D-H, Zhang J, Li J, Gao B, Ji Q, Maynard J, Ricketts S-A, Cross D, Cosulich S (2012). Preclinical Pharmacology of AZD5363, an Inhibitor of AKT: Pharmacodynamics, Antitumor Activity, and Correlation of Monotherapy Activity with Genetic Background. Molecular Cancer Therapeutics.

[236] Myers AP, Broaddus R, Makker V, Konstantinopoulos PA, Drapkin R, Horowitz NS, Liu J, Van Hummelen P, Meric-Bernstam F, Birrer MJ (2013). Phase II, two-stage, two-arm, PIK3CA mutation stratified trial of MK-2206 in recurrent endometrial cancer (EC). ASCO Annual Meeting Proceedings.

[237] Fu S, Hennessy BT, Ng CS, Ju Z, Coombes KR, Wolf JK, Sood AK, Levenback CF, Coleman RL, Kavanagh JJ (2012). Perifosine plus docetaxel in patients with platinum and taxane resistant or refractory high-grade epithelial ovarian cancer. Gynecologic oncology.

[238] Banerji U, Ranson M, Schellens JH, Esaki T, Dean E, Zivi A, Van der Noll R, Stockman PK, Marotti M, Garrett MD (2013). Abstract LB-66: Results of two phase I multicenter trials of AZD5363, an inhibitor of AKT1, 2 and 3: Biomarker and early clinical evaluation in Western and Japanese patients with advanced solid tumors. Cancer Research.

[239] Gungor H, Saleem A, Agarwal R, Blagden S, Michael A, Stronach E, Chen M, Pickford E, Rama N, Lewis Y (2011). Pharmacokinetic (PK)/pharmacodynamic (PD) analysis of escalating repeat doses of the AKT inhibitor GSK2141795 (GSK795) in patients (pts) with ovarian cancer. ASCO Annual Meeting Proceedings.

[240] Burris H, Siu L, Infante J, Wheler J, Kurkjian C, Opalinska J, Smith D, Antal J, Gauvin J, Gonzalez T (2011). Safety, pharmacokinetics (PK), pharmacodynamics (PD), and clinical activity of the oral AKT inhibitor GSK2141795 (GSK795) in a phase I first-in-human study. ASCO Annual Meeting Proceedings.

[241] Kurzrock R, Patnaik A, Rosenstein L, Fu S, Papadopoulos K, Smith D, Falchook G, Chambers G, Gauvin J, Naing A (2011). Phase I dose-escalation of the oral MEK1/2 inhibitor GSK1120212 (GSK212) dosed in combination with the oral AKT inhibitor GSK2141795 (GSK795). ASCO Annual Meeting Proceedings.

[242] Janku F, Wheler JJ, Westin SN, Moulder SL, Naing A, Tsimberidou AM, Fu S, Falchook GS, Hong DS, Garrido-Laguna I (2012). PI3K/AKT/mTOR inhibitors in patients with breast and gynecologic malignancies harboring PIK3CA mutations. Journal of Clinical Oncology.

[243] Ibrahim YH, García-García C, Serra V, He L, Torres-Lockhart K, Prat A, Anton P, Cozar P, Guzmán M, Grueso J (2012). PI3K inhibition impairs BRCA1/2 expression and sensitizes BRCA-proficient triple-negative breast cancer to PARP inhibition. Cancer discovery.

[244] Wullschleger S, Loewith R, Hall MN (2006). TOR signaling in growth and metabolism. Cell.

[245] Behbakht K, Sill MW, Darcy KM, Rubin SC, Mannel RS, Waggoner S, Schilder RJ, Cai KQ, Godwin AK, Alpaugh RK (2011). Phase II trial of the mTOR inhibitor, temsirolimus and evaluation of circulating tumor cells and tumor biomarkers in persistent and recurrent epithelial ovarian and primary peritoneal malignancies: a Gynecologic Oncology Group study. Gynecologic oncology.

[246] Takano M, Kikuchi Y, Kudoh K, Goto T, Furuya K, Kikuchi R, Kita T, Fujiwara K, Shiozawa T, Aoki D (2011). Weekly administration of temsirolimus for heavily pretreated patients with clear cell carcinoma of the ovary: a report of six cases. International journal of clinical oncology.

[247] Morgan R, Oza A, Qin R, Laumann K, Mackay H, Strevel E, Welch S, Sullivan D, Wenham R, Chen H (2011). A phase II trial of temsirolimus and bevacizumab in patients with endometrial, ovarian, hepatocellular carcinoma, carcinoid, or islet cell cancer: Ovarian cancer (OC) subset--A. study of the Princess Margaret, Mayo, Southeast phase II, and California Cancer (CCCP) N01 Consortia NCI# 8233. ASCO Annual Meeting Proceedings.

[248] Fleming GF, Filiaci VL, Marzullo B, Zaino RJ, Davidson SA, Pearl M, Makker V, Burke JJ, Zweizig SL, Van Le L (2014). Temsirolimus with or without megestrol acetate and tamoxifen for endometrial cancer: a gynecologic oncology group study. Gynecologic oncology.

[249] Oza AM, Elit L, Tsao M-S, Kamel-Reid S, Biagi J, Provencher DM, Gotlieb WH, Hoskins PJ, Ghatage P, Tonkin KS (2011). Phase II study of temsirolimus in women with recurrent or metastatic endometrial cancer: a trial of the NCIC Clinical Trials Group. Journal of Clinical Oncology.

[250] Einstein MH, Wenham RM, Morgan R, Cristea MC, Strevel EL, Oza AM, Kaubisch A, Fruth B, Qin R, Erlichman C (2012). Phase II trial of temsirolimus and bevacizumab for initial recurrence of endometrial cancer. ASCO Annual Meeting Proceedings.

[251] Alvarez EA, Brady WE, Walker JL, Rotmensch J, Zhou XC, Kendrick JE, Yamada SD, Schilder JM, Cohn DE, Harrison CR (2013). Phase II trial of combination bevacizumab and temsirolimus in the treatment of recurrent or persistent endometrial carcinoma: a Gynecologic Oncology Group study. Gynecologic oncology.

[252] Mabuchi S, Altomare DA, Connolly DC, Klein-Szanto A, Litwin S, Hoelzle MK, Hensley HH, Hamilton TC, Testa JR (2007). RAD001 (Everolimus) delays tumor onset and progression in a transgenic mouse model of ovarian cancer. Cancer Research.

[253] Mabuchi S, Altomare DA, Cheung M, Zhang L, Poulikakos PI, Hensley HH, Schilder RJ, Ozols RF, Testa JR (2007). RAD001 inhibits human ovarian cancer cell proliferation, enhances cisplatin-induced apoptosis, and prolongs survival in an ovarian cancer model. Clinical Cancer Research.

[254] Slomovitz BM, Lu KH, Johnston T, Coleman RL, Munsell M, Broaddus RR, Walker C, Ramondetta LM, Burke TW, Gershenson DM (2010). A phase 2 study of the oral mammalian target of rapamycin inhibitor, everolimus, in patients with recurrent endometrial carcinoma. Cancer.

[255] Slomovitz BM, Jiang Y, Yates MS, Soliman PT, Johnston T, Nowakowski M, Levenback C, Zhang Q, Ring K, Munsell MF (2015). Phase II study of everolimus and letrozole in patients with recurrent endometrial carcinoma. Journal of Clinical Oncology.

[256] Ray-Coquard I, Favier L, Weber B, Roemer-Becuwe C, Bougnoux P, Fabbro M, Floquet A, Joly F, Plantade A, Paraiso D (2013). Everolimus as second-or third-line treatment of advanced endometrial cancer: ENDORAD, a phase II trial of GINECO. British journal of cancer.

[257] Sarbassov DD, Guertin DA, Ali SM, Sabatini DM (2005). Phosphorylation and regulation of Akt/PKB by the rictor-mTOR complex. Science.

[258] O’Reilly KE, Rojo F, She Q-B, Solit D, Mills GB, Smith D, Lane H, Hofmann F, Hicklin DJ, Ludwig DL (2006). mTOR inhibition induces upstream receptor tyrosine kinase signaling and activates Akt. Cancer research.

[259] Leslie K, Sill M, Darcy K, Baron A, Wilken J, Godwin A, Cook L, Schilder R, Schilder J, Maihle N (2009). Efficacy and safety of gefitinib and potential prognostic value of soluble EGFR, EGFR mutations, and tumor markers in a Gynecologic Oncology Group phase II trial of persistent or recurrent endometrial cancer. ASCO Annual Meeting Proceedings.

[260] Posadas EM, Liel MS, Kwitkowski V, Minasian L, Godwin AK, Hussain MM, Espina V, Wood BJ, Steinberg SM, Kohn EC (2007). A phase II and pharmacodynamic study of gefitinib in patients with refractory or recurrent epithelial ovarian cancer. Cancer.

[261] Schilder RJ, Sill MW, Chen X, Darcy KM, Decesare SL, Lewandowski G, Lee RB, Arciero CA, Wu H, Godwin AK (2005). Phase II study of gefitinib in patients with relapsed or persistent ovarian or primary peritoneal carcinoma and evaluation of epidermal growth factor receptor mutations and immunohistochemical expression: a Gynecologic Oncology Group Study. Clinical Cancer Research.

[262] Wagner U, du Bois A, Pfisterer J, Huober J, Loibl S, Lück H-J, Sehouli J, Gropp M, Stähle A, Schmalfeldt B (2007). Gefitinib in combination with tamoxifen in patients with ovarian cancer refractory or resistant to platinum–taxane based therapy—a phase II trial of the AGO Ovarian Cancer Study Group (AGO-OVAR 2.6). Gynecologic oncology.

[263] Pautier P, Joly F, Kerbrat P, Bougnoux P, Fumoleau P, Petit T, Rixe O, Ringeisen F, Carrasco AT, Lhommé C (2010). Phase II study of gefitinib in combination with paclitaxel (P) and carboplatin (C) as second-line therapy for ovarian, tubal or peritoneal adenocarcinoma (1839IL/0074). Gynecologic oncology.

[264] Mavroudis D, Efstathiou E, Polyzos A, Athanasiadis A, Milaki G, Kastritis E, Kalykaki A, Saridaki Z, Dimopoulos A, Georgoulias V (2004). A phase I-II trial of gefitinib in combination with vinorelbine and oxaliplatin as salvage therapy in women with advanced ovarian cancer (AOC). ASCO Annual Meeting Proceedings.

[265] Oza AM, Eisenhauer EA, Elit L, Cutz J-C, Sakurada A, Tsao MS, Hoskins PJ, Biagi J, Ghatage P, Mazurka J (2008). Phase II study of erlotinib in recurrent or metastatic endometrial cancer: NCIC IND-148. Journal of Clinical Oncology.

[266] Finkler N, Gordon A, Crozier M, Edwards R, Figueroa J, Garcia A, Hainsworth J, Irwin D, Silberman S, Allen L (2001). Phase 2 evaluation of OSI-774, a potent oral antagonist of the EGFR-TK in patients with advanced ovarian carcinoma. Proc Am Soc Clin Oncol.

[267] Vasey P, Gore M, Wilson R, Rustin G, Gabra H, Guastalla J, Lauraine E, Paul J, Carty K, Kaye S (2008). A phase Ib trial of docetaxel, carboplatin and erlotinib in ovarian, fallopian tube and primary peritoneal cancers. British journal of cancer.

[268] Hirte H, Oza A, Swenerton K, Ellard S, Grimshaw R, Fisher B, Tsao M, Seymour L (2010). A phase II study of erlotinib (OSI-774) given in combination with carboplatin in patients with recurrent epithelial ovarian cancer (NCIC CTG IND. 149). Gynecologic oncology.

[269] Seiden MV, Burris H, Matulonis U, Hall J, Armstrong D, Speyer J, Weber J, Muggia F (2007). A phase II trial of EMD72000 (matuzumab), a humanized anti-EGFR monoclonal antibody, in patients with platinum-resistant ovarian and primary peritoneal malignancies. Gynecologic oncology.

[270] Schilder RJ, Pathak HB, Lokshin AE, Holloway RW, Alvarez RD, Aghajanian C, Min H, Devarajan K, Ross E, Drescher CW (2009). Phase II trial of single agent cetuximab in patients with persistent or recurrent epithelial ovarian or primary peritoneal carcinoma with the potential for dose escalation to rash. Gynecologic oncology.

[271] Konner J, Schilder RJ, DeRosa FA, Gerst SR, Tew WP, Sabbatini PJ, Hensley ML, Spriggs DR, Aghajanian CA (2008). A phase II study of cetuximab/paclitaxel/carboplatin for the initial treatment of advanced-stage ovarian, primary peritoneal, or fallopian tube cancer. Gynecologic oncology.

[272] Secord AA, Blessing JA, Armstrong DK, Rodgers WH, Miner Z, Barnes MN, Lewandowski G, Mannel RS (2008). Phase II trial of cetuximab and carboplatin in relapsed platinum-sensitive ovarian cancer and evaluation of epidermal growth factor receptor expression: a Gynecologic Oncology Group study. Gynecologic oncology.

[273] Takahashi K, Saga Y, Mizukami H, Takei Y, Machida S, Fujiwara H, Ozawa K, Suzuki M (2009). Cetuximab inhibits growth, peritoneal dissemination, and lymph node and lung metastasis of endometrial cancer, and prolongs host survival. International journal of oncology.

[274] Rose PG, Brunetto VL, VanLe L, Bell J, Walker JL, Lee RB (2000). A phase II trial of anastrozole in advanced recurrent or persistent endometrial carcinoma: a Gynecologic Oncology Group study. Gynecologic oncology.

[275] Del Carmen MG, Fuller AF, Matulonis U, Horick NK, Goodman A, Duska LR, Penson R, Campos S, Roche M, Seiden MV (2003). Phase II trial of anastrozole in women with asymptomatic müllerian cancer. Gynecologic oncology.

[276] Krasner C, Debernardo R, Findley M, Penson R, Matulonis U, Atkinson T, Roche M, Seiden M (2005). Phase II trial of anastrazole in combination with gefitinib in women with asymptomatic mullerian cancer. ASCO Annual Meeting Proceedings.

[277] Ma B, Oza A, Eisenhauer E, Stanimir G, Carey M, Chapman W, Latta E, Sidhu K, Powers J, Walsh W (2004). The activity of letrozole in patients with advanced or recurrent endometrial cancer and correlation with biological markers–a study of the National Cancer Institute of Canada Clinical Trials Group. International Journal of Gynecological Cancer.

[278] Bowman A, Gabra H, Langdon SP, Lessells A, Stewart M, Young A, Smyth JF (2002). CA125 Response Is Associated with Estrogen Receptor Expression in a Phase II Trial of Letrozole in Ovarian Cancer Identification of an Endocrine-sensitive Subgroup. Clinical cancer research.

[279] Papadimitriou CA, Markaki S, Siapkaras J, Vlachos G, Efstathiou E, Grimani I, Hamilos G, Zorzou M, Dimopoulos M-A (2004). Hormonal therapy with letrozole for relapsed epithelial ovarian cancer. Oncology.

[280] Smyth JF, Gourley C, Walker G, MacKean MJ, Stevenson A, Williams AR, Al Nafussi A, Rye T, Rye R, Stewart M (2007). Antiestrogen therapy is active in selected ovarian cancer cases: the use of letrozole in estrogen receptor–positive patients. Clinical Cancer Research.

[281] Ramirez PT, Schmeler KM, Milam MR, Slomovitz BM, Smith JA, Kavanagh JJ, Deavers M, Levenback C, Coleman RL, Gershenson DM (2008). Efficacy of letrozole in the treatment of recurrent platinum-and taxane-resistant high-grade cancer of the ovary or peritoneum. Gynecologic oncology.

[282] Verma S, Alhayki M, Baines K, Le T, Rambout L, Hopkins L, Fung Kee Fung M (2006). Phase II study of exemestane (E) in refractory ovarian cancer (ROC). ASCO Annual Meeting Proceedings.

[283] Covens AL, Filiaci V, Gersell D, Lutman CV, Bonebrake A, Lee Y-C (2011). Phase II study of fulvestrant in recurrent/metastatic endometrial carcinoma: a Gynecologic Oncology Group study. Gynecologic oncology.

[284] Argenta PA, Thomas SG, Judson PL, Downs LS, Geller MA, Carson LF, Jonson AL, Ghebre R (2009). A phase II study of fulvestrant in the treatment of multiply-recurrent epithelial ovarian cancer. Gynecologic oncology.

[285] Argenta PA, Um I, Kay C, Harrison D, Faratian D, Sueblinvong T, Geller MA, Langdon SP (2013). Predicting response to the anti-estrogen fulvestrant in recurrent ovarian cancer. Gynecologic oncology.

[286] Burke TW, Walker CL (2003). Arzoxifene as therapy for endometrial cancer. Gynecologic oncology.

[287] McMeekin DS, Gordon A, Fowler J, Melemed A, Buller R, Burke T, Bloss J, Sabbatini P (2003). A phase II trial of arzoxifene, a selective estrogen response modulator, in patients with recurrent or advanced endometrial cancer. Gynecologic oncology.

[288] Mäenpää J, SipilÄ P, Kangas L, Karnani P, Grönroos M (1992). Chemosensitizing effect of an antiestrogen, toremifene, on ovarian cancer. Gynecologic oncology.

[289] Slomovitz B, Brown J, Johnston T, Mura D, Levenback C, Wolf J, Adler K, Lu K, Coleman R (2011). A phase II study of everolimus and letrozole in patients with recurrent endometrial carcinoma. ASCO Annual Meeting Proceedings.

[290] Brasseur K, Leblanc V, Fabi F, Parent S, Descôteaux C, Bérubé G, Asselin E (2013). ERα-Targeted Therapy in Ovarian Cancer Cells by a Novel Estradiol-Platinum(II) Hybrid. Endocrinology.

[291] Leffers N, Lambeck AJ, Gooden MJ, Hoogeboom BN, Wolf R, Hamming IE, Hepkema BG, Willemse PH, Molmans BH, Hollema H (2009). Immunization with a P53 synthetic long peptide vaccine induces P53‐specific immune responses in ovarian cancer patients, a phase II trial. International Journal of Cancer.

[292] Vermeij R, Leffers N, Hoogeboom BN, Hamming IL, Wolf R, Reyners AK, Molmans BH, Hollema H, Bart J, Drijfhout JW (2012). Potentiation of a p53‐SLP vaccine by cyclophosphamide in ovarian cancer: A single‐arm phase II study. International Journal of Cancer.

[293] Leffers N, Vermeij R, Hoogeboom BN, Schulze UR, Wolf R, Hamming IE, van der Zee AG, Melief KJ, van der Burg SH, Daemen T (2012). Long‐term clinical and immunological effects of p53‐SLP® vaccine in patients with ovarian cancer. International Journal of Cancer.

[294] Buller RE, Runnebaum IB, Karlan BY, Horowitz JA, Shahin M, Buekers T, Petrauskas S, Kreienberg R, Slamon D, Pegram M (2002). A phase I/II trial of rAd/p53 (SCH 58500) gene replacement in recurrent ovarian cancer. Cancer gene therapy.

[295] Buller RE, Shahin MS, Horowitz JA, Runnebaum IB, Mahavni V, Petrauskas S, Kreienberg R, Karlan B, Slamon D, Pegram M (2002). Long term follow-up of patients with recurrent ovarian cancer after Ad p53 gene replacement with SCH 58500. Cancer gene therapy.

[296] Heise C, Sampson-Johannes A, Williams A, Mccormick F, Von Hoff DD, Kirn DH (1997). ONYX-015, an E1B gene-attenuated adenovirus, causes tumor-specific cytolysis and antitumoral efficacy that can be augmented by standard chemotherapeutic agents. Nature medicine.

[297] Vasey P, Shulman L, Campos S, Davis J, Gore M, Johnston S, Kirn D, O’neill V, Siddiqui N, Seiden MV (2002). Phase I Trial of Intraperitoneal Injection of the E1B-55-kd-Gene–Deleted Adenovirus ONYX-015 (dl1520) given on days 1 through 5 every 3 weeks in patients with recurrent/refractory epithelial ovarian cancer. Journal of clinical oncology.

[298] Khuri FR, Nemunaitis J, Ganly I, Arseneau J, Tannock IF, Romel L, Gore M, Ironside J, MacDougall R, Heise C (2000). A controlled trial of intratumoral ONYX-015, a selectively-replicating adenovirus, in combination with cisplatin and 5-fluorouracil in patients with recurrent head and neck cancer. Nature medicine.

[299] Reid T, Galanis E, Abbruzzese J, Sze D, Wein LM, Andrews J, Randlev B, Heise C, Uprichard M, Hatfield M (2002). Hepatic arterial infusion of a replication-selective oncolytic adenovirus (dl1520) phase ii viral, immunologic, and clinical endpoints. Cancer research.

[300] Hirai H, Iwasawa Y, Okada M, Arai T, Nishibata T, Kobayashi M, Kimura T, Kaneko N, Ohtani J, Yamanaka K (2009). Small-molecule inhibition of Wee1 kinase by MK-1775 selectively sensitizes p53-deficient tumor cells to DNA-damaging agents. Molecular cancer therapeutics.

[301] Brana I, Moore KN, Shapira-Frommer R, Welch S, Jou Y-M, Marinucci M, Freshwater T, Rose S, Oza AM (2013). Targeting p53 mutant ovarian cancer: Phase I results of the WEE1 inhibitor MK-1775 with carboplatin plus paclitaxel in patients (pts) with platinum-sensitive, p53-mutant ovarian cancer (OC). ASCO Annual Meeting Proceedings.

[302] Oza AM, Weberpals JI, Provencher DM, Grischke E-M, Hall M, Uyar D, Estevez-Diz MDP, Marme F, Kuzmin A, Rosenberg P (2015). An international, biomarker-directed, randomized, phase II trial of AZD1775 plus paclitaxel and carboplatin (P/C) for the treatment of women with platinum-sensitive, TP53-mutant ovarian cancer. ASCO Annual Meeting Proceedings.

[303] Leijen S, Van Geel R, Sonke GS, de Jong D, Rosenberg EH, Marchetti S, Pluim D, van Werkhoven ED, Rose S, Lee MA (2015). Phase II study with Wee1 inhibitor AZD1775 plus carboplatin in patients with p53 mutated ovarian cancer refractory or resistant (< 3 months) to standard first line therapy. ASCO Annual Meeting Proceedings.

[304] Lheureux S, Weberpals JI, Wahner Hendrickson AE, Fleming GF, Olawaiye A, Brana I, Mackay H, Dhani NC, Wilson MK, Rodriguez-Freixinos V (2015). A randomized, placebo-controlled phase II trial comparing gemcitabine monotherapy to gemcitabine in combination with AZD 1775 (MK 1775) in women with recurrent, platinum-resistant epithelial ovarian, primary peritoneal, or Fallopian tube cancers: Trial of Princess Margaret,. and California consortia. ASCO Annual Meeting Proceedings.

[305] Kobayashi N, Abedini M, Sakuragi N, Tsang BK (2013). PRIMA-1 increases cisplatin sensitivity in chemoresistant ovarian cancer cells with p53 mutation: a requirement for Akt down-regulation. Journal of ovarian research.

[306] Mohell N, Alfredsson J, Å Fransson, Uustalu M, Byström S, Gullbo J, Hallberg A, Bykov V, Björklund U, Wiman K (2015). APR-246 overcomes resistance to cisplatin and doxorubicin in ovarian cancer cells. Cell death & disease.

[307] Mikael vE, Klas GW, Hani G, James DB, Ignace V, Charlie G, Smith A, Jessica A, Nina M, John G (2014). Phase I/II study of APR-246, a mutant p53 reactivating compound, in combination with standard chemotherapy in platinum sensitive ovarian cancer. 12th international congress on targeted anticancer therapies.

[308] Kline CL, Shanmugavelandy SS, Kester M, Irby RB (2009). Delivery of PAR-4 plasmid in vivo via nanoliposomes sensitizes colon tumor cells subcutaneously implanted into nude mice to 5-FU. Cancer biology & therapy.

[309] Burikhanov R, Yanming Z, Goswami A, Qiu S, Schwarze SR, Rangnekar VM (2009). The Tumor Suppressor Par-4 Activates an Extrinsic Pathway for Apoptosis.

[310] Sarkar S, Jain S, Rai V, Sahoo DK, Raha S, Suklabaidya S, Senapati S, Rangnekar VM, Maiti IB, Dey N (2015). Plant-derived SAC domain of PAR-4 (Prostate Apoptosis Response 4) exhibits growth inhibitory effects in prostate cancer cells. Frontiers in plant science.

[311] Chakraborty M, Qiu SG, Vasudevan KM, Rangnekar VM (2001). Par-4 Drives Trafficking and Activation of Fas and FasL to Induce Prostate Cancer Cell Apoptosis and Tumor Regression. Cancer Research.

[312] Klintman M, Buus R, Cheang MCU, Sheri A, Smith IE, Dowsett M (2016). Changes in Expression of Genes Representing Key Biologic Processes after Neoadjuvant Chemotherapy in Breast Cancer, and Prognostic Implications in Residual Disease. Clinical Cancer Research.

[313] Burrell RA, McGranahan N, Bartek J, Swanton C (2013). The causes and consequences of genetic heterogeneity in cancer evolution. Nature.

[314] Zhao J (2016). Cancer stem cells and chemoresistance: The smartest survives the raid. Pharmacology & therapeutics.

[315] Abdullah LN, Chow EK-H (2013). Mechanisms of chemoresistance in cancer stem cells. Clinical and translational medicine.

[316] Haygood CLW, Arend RC, Straughn JM, Buchsbaum DJ (2014). Ovarian cancer stem cells: Can targeted therapy lead to improved progression-free survival?. World J Stem Cells.

[317] Bapat SA, Mali AM, Koppikar CB, Kurrey NK (2005). Stem and progenitor-like cells contribute to the aggressive behavior of human epithelial ovarian cancer. Cancer research.

[318] Hu L, McArthur C, Jaffe R (2010). Ovarian cancer stem-like side-population cells are tumourigenic and chemoresistant. British journal of cancer.

[319] Zhang S, Balch C, Chan MW, Lai H-C, Matei D, Schilder JM, Yan PS, Huang TH, Nephew KP (2008). Identification and characterization of ovarian cancer-initiating cells from primary human tumors. Cancer research.

[320] Ma S, Lee T, Zheng B, Chan K, Guan X (2008). CD133+; HCC cancer stem cells confer chemoresistance by preferential expression of the Akt/PKB survival pathway. Oncogene.

[321] Tomida A, Tsuruo T (1999). Drug resistance mediated by cellular stress response to the microenvironment of solid tumors. Anti-cancer drug design.

[322] Brown JM, Wilson WR (2004). Exploiting tumour hypoxia in cancer treatment. Nature Reviews Cancer.

[323] Balkwill FR, Capasso M, Hagemann T (2012). The tumor microenvironment at a glance. Journal of cell science.

