# Supplementary material for: Chemoresistance and targeted therapies in ovarian and endometrial cancers
Source: Oncotarget. 2016 Dec 19;8(3):4008–42. doi: 10.18632/oncotarget.14021 (PMC5354810; doi:10.18632/oncotarget.14021)
Supplement: Supplementary file 1 [file oncotarget-08-4008-s001.docx]

Chemoresistance and targeted therapies in ovarian and endometrial cancers

**Supplementary Material**

**Supplementary Table 1:**  Clinical trials of targeted therapies in ovarian and endometrial cancers

The table summarizes all the clinical trials discussed in the manuscript. The different column indicates the treatment, if it has been combined with a chemotherapeutic agent, the target, the clinical trial phase, the tissues studied, the number of patients evaluated, the responses observed, additional comments as well as the bibliographical reference to the clinical trial.

| Agent | Single/combined | Target | Phase | Tissue | # patients | Response | Comments | Reference |
| --- | --- | --- | --- | --- | --- | --- | --- | --- |
| Tariquidar | Combined with docetaxel | p-Glycoprotein and mitosis |  | Ovary, cervix and lung | 48 patients | 4/48 PR (1 PR in ovarian cancer) |  | [183] |
| Olaparib | Single | PARP | II | Ovary | 33 patients | 11/33 ORR |  | [187] |
| Olaparib | Single | PARP | II | Ovary | 265 patients platinum-sensitive (136 treated and 129 placebo) | Treated patients: PFS of 8,4 months Placebo patients: PFS of 4,8 months | The effect observed was independent of BRCA status. | [188] |
| Olaparib | Single | PARP | II | Ovary | 63 patients (17 BRCA mutations and 46 BRCA WT) | BRCA mutations: 7/17 ORR  BRCA WT: 11/46 ORR |  | [189] |
| Olaparib | Single | PARP | II | Ovary | 193 patients platinum-resistant | 60/193 ORR |  | [190] |
| Olaparib | Combined with carboplatin | PARP and mitosis | I/Ib | Ovary | 37 patients | 1/37 CR; 15/37 PR | FOXO3a expression was predictive of the response. | [191] |
| Olaparib | Combined with carboplatin and paclitaxel | PARP and mitosis | II | Ovary | 162 patients platinum-sensitive (81 treated with Olaparib+chemotherapy and 75 chemotherapy only) | PFS of 12,2 months for combined treatment versus 9,6 months for chemotherapy only | Only 41/107 patients had BRCA mutations. | [193] |
| Olaparib | Combined with cediranib | PARP and angiogenesis | II | Ovary | 90 patients platinum-sensitive (44 treated with Olaparib+cediranib and 46 Olaparib only) | PFS of 17,7 months for combined treatment versus 9 months for Olaparib only |  | [194] |
| Olaparib | Single | PARP |  | Endometrium | 58-year-old women | Reduction of brain metastases Improvement of tumor-related symptoms | Absence of BRCA mutations but instead, PTEN loss. | [206] |
| Veliparib | Single | PARP | II | Ovary | 50 patients (20 platinum-sensitive and 30 platinum-resistant) | All patients: ORR of 26% (2 CR and 11 PR) Platinum-sensitive: ORR of 35% Platinum-resistant: ORR of 20% |  | [195] |
| Veliparib | Combined with cyclophosphamide | PARP and mitosis | II | Ovary | 72 patients (37 treated with Veliparib+cyclophosphamide and 38 cyclophosphamide only) | Combination: 1/37 CR; 3/37 PR Cyclophosphamide only: 1/38 CR; 6/37 PR | The addition of Veliparib did not improve the response rate or PFS. | [196] |
| Niraparib | Single | PARP | I | Ovary and peritoneum | 42 patients (20 BRCA mutations and 22 BRCA WT) | BRCA mutations : 8/20 PR; 2/20 SD  BRCA WT : 5/22 PR; 3/22 SD | Platinum sensitivity of patients was also considered when analyzing the response rate. No significant difference was observed in the CBR from BRCA mutation carrier (50% for sensitive and 50% for resistant), however, CBR was twice lower in BRCA WT platinum resistant patients (67% for sensitive and 32% for resistant)  No correlation has been associated with loss of PTEN. | [197] |
| Iniparib | Combined with carboplatin and gemcitabine | PARP and mitosis | II | Ovary | 17 patients platinum-sensitive | ORR of 71% | BRCA status was not associated with response rate. | [198] |
| Iniparib | Combined with carboplatin and paclitaxel | PARP and mitosis |  | Uterus | 17 patients | 4/17 ORR |  | [199] |
| Rucaparib | Single | PARP | I | Diverse (including ovary) | 29 patients (7 ovarian/peritoneal cancer) | All cancer : 2/29 PR; 10/29 SD  Ovarian/peritoneal cancer: 1/7 PR; 5/7 SD; 6/7 CBR (86%) | Efficient in ovarian/peritoneal cancer patients. | [201] |
| Rucaparib | Single | PARP | II | Ovary | 22 patients | ORR of 15% | 12 of the 13 patients who received continuously Rucaparib achieved either CR, PR or SD for more than 12 weeks. | [202] |
| Decitabine | Combined with carboplatin | Demethylating genes related to MMR deficiency and mitosis | I | Ovary | 10 patients platinum-resistant | 1/10 CR; 3/10 SD | HOXA11 and BRCA1 cancer associated genes were demethylated after treatment. | [213] |
| Decitabine | Combined with carboplatin | Demethylating genes related to MMR deficiency and mitosis | II | Ovary | 17 patients platinum-resistant | Response rate of 35%  53% had a PFS > 6 months The PFS had a median of 10.2 months | Efficient demethylation of RASSF1A, HOXA10, HOXA11 and MLH1 which correlated with PFS. | [214] |
| Temsirolimus | Single | mTORC1 | II | Ovary | 54 patients | 9/54 PR |  | [245] |
| Temsirolimus | Single | mTORC1 |  | Ovary | 5 patients chemo-resistant | 1/5 PR; 1/5 SD |  | [246] |
| Temsirolimus | Combined with bevacizumab | mTORC1 and angiogenesis |  | Ovary | 31 patients (17 platinum-sensitive and 14 platinum-resistant); *25 patients were evaluable* | 3/25 PR; 9/25 SD | The 3 PR observed were from the platinum-resistant group. | [247] |
| Temsirolimus | Single | mTORC1 | II | Endometrium | 50 patients (29 chemo-naïve and 21 chemo-treated) | All patients: ORR of 22%  Chemo-naïve: ORR of 24% Chemo-treated: ORR of 19% |  | [248] |
| Temsirolimus | Combined with megestrol acetate and tamoxifen | mTORC1, PR and ER | II | Endometrium | 21 patients | 3/21 responses | Adding megestrol acetate and tamoxifen to temsirolimus treatment did not improve the treatment efficiency and was associated with an increased toxicity. The trial was closed early because of toxicity. | [248] |
| Temsirolimus | Single | mTORC1 | II | Endometrium | 54 patients (29 chemo-naïve and 25 chemo-treated) | Chemo-naïve: 4/29 PR; 20/29 SD Chemo-treated: 1/25 PR; 12/25 SD | No association with PTEN status. | [249] |
| Temsirolimus | Combined with bevacizumab | mTORC1 and angiogenesis | II | Endometrium | 26 patients | 5/26 PR 12/26 patients had a PFS > 6 months |  | [250] |
| Temsirolimus | Combined with bevacizumab | mTORC1 and angiogenesis | II | Endometrium | 49 patients | 1/49 CR; 11/49 PR 23/49 patients had a PFS > 6 months |  | [251] |
| Everolimus | Single | mTORC1 | II | Endometrium | 28 patients previously chemo-treated | 6/28 SD  6/28 CBR (22%) |  | [254] |
| Everolimus | Combined with Letrozole | mTORC1 and aromatase | II | Endometrium | 35 patients | 11/35 CR; 2/35 PR; 1/35 SD  14/35 CBR (40%) | Adding aromatase inhibitor increased the CBR from 22% to 40%. | [255] |
| Everolimus | Single | mTORC1 | II | Endometrium | 44 patients (2/3 previously chemo-treated) | 4/44 PR | 36% had a non-progressive disease after 3 months. | [256] |
| Buparlisib | Combined with Olaparib | Pan-PI3K and PARP | I | Ovary and breast | 34 patients (25 ovarian cancer) | Evidence of clinical benefit by RECIST 1.1 was observed on all escalating dose levels | All dose combinations allowed the observation of clinical benefits among the patients. 26 patients had BRCA mutations. | [217] |
| GDC-041 | Single | Pan-PI3K | I | Diverse (including ovary and endometrium) | 49 patients | 2/49 PR | One of the PR was an endocervical tumor with mutations on PIK3CA. CA125 responses were also observed in three patients with ovarian cancer including one with known high PIK3CA gene copy number. | [218] |
| GDC-041 | Single | Pan-PI3K | I | Diverse (including ovary) | 42 patients | 2/42 PR | One of the PR was an ovarian tumor with PTEN loss. | [219] |
| Pilaralisib | Single | Pan-PI3K | II | Endometrium | 67 patients | 2/67 CR; 2/67 PR | No association was made between the molecular alterations of the PI3K pathway. | [220] |
| BYL719 | Single | PIK3CA | I | Diverse (including ovary and endometrium) | 36 patients PI3KCA mutants | 7/36 PR | Among the PR, one from cervical, one from endometrium and one from the ovary. | [221] |
| Buparlisib | Combined with Trametinib | Pan-PI3K and MEK | Ib | Diverse (including ovary) | 113 patients (21 ovarian cancer) | All cancer patients: ORR of 6%  Ovarian cancer patients:  1/21 CR; 5/21 PR; 10/21 SD  ORR of 29% | 19 ovarian cancer patients were KRAS mutated demonstrating an efficient way to overcome this mutation. Buparlisib was efficient almost exclusively in ovarian cancer, the exception being a PR observed in a KRAS mutated NSCLC patient. | [224] |
| BYL719 | Combined with binimetinib | PIK3CA and MEK | Ib | Diverse (including ovary and endometrium) | 58 patients with RAS and BRAF mutations | 5/58 PR; 18/58 SD | Four patients with ovarian cancer had KRAS mutation and 3 of these had a PR. A patient with endometrial cancer and KRAS mutation also had a PR. | [225] |
| MK-2206 | Single | AKT | II | Endometrium | 36 patients (9 PIK3CA mutants and 27 PIK3CA WT) | 2/36 PR | Results were independent of the PIK3CA status. | [236] |
| Perifosine | Combined with docetaxel | AKT and mitosis | I | Ovary | 21 patients taxane-resistants | 1/21 PR; 3/21 SD | The patient with a PR was PTEN mutant. Among the patients with SD, two were PIK3CA mutant and 1 WT. Patients with KRAS mutation had a rapid tumor progression. | [237] |
| AZD5363 | Single | AKT | I | Diverse (including ovary and endometrium) | 92 patients | 2/92 PR; 1/92 SD | Among the PR, one endometrioid cancer of the ovary and one cervical cancer with either PIK3CA or AKT1 mutation. The patient with a SD was an endometrioid cancer of the ovary with PIK3CA mutations. | [238] |
| GSK2141795 | Single | AKT | I | Ovary | 12 patients platinum-resistant | 8/12 SD | The four patients left had a progressive disease. | [239] |
| GSK2141795 | Single | AKT | I | Diverse (including endometrium) | 66 patients (12 endometrial cancer) | Endometrial cancer patients:  2/12 SD | The two patients with SD were PIK3CA mutant and/or PTEN loss. | [240] |
| GSK2141795 | Combined with GSK1120212 | AKT and MEK | I | Diverse (including ovary and endometrium) | 13 patients | 3/13 had weak tumor regression | The patients with SD were two patients with ovarian cancer and one with endometrial cancer. | [241] |
| Gefitinib | Single | EGFR | II | Endometrium | 26 patients | 1/26 CR; 7/26 PR 4/26 patients had a PFS > 6 months |  | [259] |
| Gefitinib | Single | EGFR | II | Ovary | 24 patients | 9/24 SD > 2 months |  | [260] |
| Gefitinib | Single | EGFR | II | Ovary or peritoneum | 27 patients | 1/27 ORR 4/27 patients had a PFS > 6 months |  | [261] |
| Gefitinib | Combined with tamoxifen | EGFR and ER | II | Ovary | 56 patients platinum- and taxane- resistant | 16/56 SD |  | [262] |
| Gefitinib | Combined with paclitaxel and carboplatin | EGFR and mitosis | II | Ovary, fallopian tube or peritoneum | 68 patients (26 platinum-resistant and 42 platinum-sensitive) | Platinum-resistant: ORR of 19.2% and CBR of 69.2%  Platinum-sensitive: ORR of 61.9% and CBR of 81.0% |  | [263] |
| Gefitinib | Combined with oxaliplatin and vinorelbine | EGFR and mitosis | I/II | Ovary | 33 patients (23 platinum-resistant and 10 platinum-sensitive) | Platinum-resistant: ORR of 23,8% (3/26 CR; 2/26 PR) Platinum-sensitive: ORR of 90% (4/10 CR; 5/10 PR) |  | [264] |
| Erlotinib | Single | EGFR | II | Endometrium | 32 patients | 4/32 PR; 15/32 SD |  | [265] |
| Erlotinib | Single | EGFR | II | Ovary | 34 patients | 2/34 PR; 14/34 SD |  | [266] |
| Erlotinib | Combined with carboplatin and docetaxel | EGFR and mitosis | Ib | Ovary | 23 patients chemo-naïve | ORR of 52% (5/23 CR; 7/23 PR) |  | [267] |
| Erlotinib | Combined with carboplatin | EGFR and mitosis | II | Ovary | 50 patients (17 platinum-resistant and 33 platinum-sensitive) | Platinum-resistant: 1/17 PR Platinum-sensitive: 14/33 PR |  | [268] |
| Matuzumab | Single | EGFR | II | Ovary | 37 patients platinum-resistant | 7/37 SD |  | [269] |
| Cetuximab | Single | EGFR | II | Ovary or peritoneum | 25 patients platinum-resistant | 1/25 PR; 9/25 SD |  | [270] |
| Cetuximab | Combined with carboplatin and paclitaxel | EGFR and mitosis | II | Ovary | 40 patients chemo-naïve | No increase of PFS by adding Cetuximab |  | [271] |
| Cetuximab | Combined with carboplatin | EGFR and mitosis | II | Ovary | 28 patients platinum-sensitive | 9/28 ORR (3/28 CR; 6/28 PR); 6/28 SD | 26 patients had EGFR-positive tumors. | [272] |
| Anastrozole | Single | Aromatase | II | Endometrium | 23 patients | 2/23 PR; 2/23 SD |  | [274] |
| Anastrozole | Single | Aromatase | II | Ovary, fallopian tube or peritoneum | 53 patients (43 ovarian cancer) | 1/53 PR; 36/53 SD |  | [275] |
| Anastrozole | Combined with gefitinib | Aromatase and EGFR | II | Ovary, fallopian tube or peritoneum | 35 patients (30 ovarian cancer) *23 patients were evaluable* | 1/23 CR; 14/23 SD |  | [276] |
| Letrozole | Single | Aromatase | II | Endometrium | 28 patients chemo-naïve | 1/28 CR; 2/28 PR; 11/28 SD | Different markers including the hormone receptors were screened but were not correlated with response to letrozole. | [277] |
| Letrozole | Single | Aromatase | II | Ovary | 50 patients | 10/50 SD | They observed a correlation between the response to letrozole and high estrogen receptor level. | [278] |
| Letrozole | Single | Aromatase | II | Ovary | 21 patients | 1/21 CR; 2/21 PR; 4/21 SD | No association was found between hormone receptors and response to letrozole. | [279] |
| Letrozole | Single | Aromatase | II | Ovary | 33 patients expressing ER | 3/33 PR; 14/33 SD |  | [280] |
| Letrozole | Single | Aromatase | II | Ovary | 31 patients platinum- and taxane- resistant; expressing ER | 7/31 SD |  | [281] |
| Exemestane | Single | Aromatase | II | Ovary | 24 patients platinum- and taxane- treated | 8/24 SD |  | [282] |
| Fulvestrant | Single | Estrogen receptor | II | Endometrium | 53 patients (31 ER positive and 22 ER negative) | ER positive: 1/31 CR; 4/31 PR; 9/31 SD ER negative: 4/22 SD |  | [283] |
| Fulvestrant | Single | Estrogen receptor | II | Ovary | 26 patients | 1/26 CR; 1/26 PR; 9/26 SD | Response rate was positively correlated with ER status. | [284] |
| Arzoxifene | Single | Estrogen receptor | II | Endometrium | 66 patients (35 progestogen sensitive and 31 progestogen refractory) | ORR of 25% (ORR of 34% in the progestogen sensitive group) |  | [286] |
| Arzoxifene | Single | Estrogen receptor | II | Endometrium | 34 patients | ORR of 31% |  | [286] |
| Arzoxifene | Single | Estrogen receptor | II | Endometrium | 29 patients chemo-naïve; expressing ER | ORR of 31% (1/29 CR; 8/29 PR) |  | [287] |
| Toremifene | Single | Estrogen receptor |  | Ovary or uterus | 8 patients | 3/8 PR; 3/8 SD | The other 2 patients had progressive disease. | [288] |
| P53-SLP | Single | P53 | II | Ovary | 20 patients | 2/20 SD | Well tolerated, stimulated T-Cell responses. | [291] |
| P53-SLP | Single, pre-treated with cyclophosphamide | P53 | II | Ovary | 10 patients | 2/10 SD | Higher number of IFN-γ-producing T cells when compared to their previous study. | [292] |
| P53-SLP | Single | P53 | II | Ovary | 20 patients | 2/20 SD (before chemotherapy) | P53-SLP did not enhance the efficiency of chemotherapy treatments, thus was not able to overcome the chemoresistance of advanced ovarian cancer patients. | [293] |
| SCH-58500 | Single | P53 | I/II | Ovary | 24 patients *16 patients evaluable* | 8/16 had at least 50% decrease of CA125 | Patient who received a single dose of SCH-58500 had a median survival of 5 months versus 13 months for those who received multiple doses of SCH-58500. | [294, 295] |
| ONYX-015 | Single | P53 | I | Ovary | 16 patients | No response |  | [297] |
| MK-1775 | Combined with carboplatin and paclitaxel | WEE1 and mitosis | I | Ovary | 14 patients platinum-sensitive | 11 PR; 3 SD |  | [301] |
| MK-1775 | Combined with carboplatin and paclitaxel | WEE1 and mitosis | II | Ovary | 121 patients platinum-sensitive (59 treated with MK-1775+chemotherapy and 61 chemotherapy only) | Combined treatment: ORR of 81% Chemotherapy only: ORR of 74% | Progression-free survival was greater with the addition of MK-1775. | [302] |
| MK-1775 | Combined with carboplatin | WEE1 and mitosis | II | Ovary | 22 patients platinum-resistant | 6/22 PR; 9/22 SD PFS of 11 months |  | [303] |

**Supplementary table 1 – Clinical trials of targeted therapies in gynecological cancers**

| **Unless specified, the column # patients indicate the number of patients evaluated* | | | |  |
| --- | --- | --- | --- | --- |
| *CR = Complete response* | *PR = Partial response* | *SD = Stable disease* | *PFS = Progression free survival* |  |
| *ORR = Objective response rate (CR+PR)* | |  |  |  |
| *CBR = Clinical benefit rate (CR+PR+SD)* | |  |  |  |
